# Accurate cancer phenotype prediction with AKLIMATE, a stacked kernel learner integrating multimodal genomic data and pathway knowledge

**DOI:** 10.1371/journal.pcbi.1008878

**Published:** 2021-04-16

**Authors:** Vladislav Uzunangelov, Christopher K. Wong, Joshua M. Stuart

**Affiliations:** Department of Biomolecular Engineering, University of California, Santa Cruz, California, United States of America; Queen’s University, CANADA

## Abstract

Advancements in sequencing have led to the proliferation of multi-omic profiles of human cells under different conditions and perturbations. In addition, many databases have amassed information about pathways and gene “signatures”—patterns of gene expression associated with specific cellular and phenotypic contexts. An important current challenge in systems biology is to leverage such knowledge about gene coordination to maximize the predictive power and generalization of models applied to high-throughput datasets. However, few such integrative approaches exist that also provide interpretable results quantifying the importance of individual genes and pathways to model accuracy. We introduce AKLIMATE, a first kernel-based stacked learner that seamlessly incorporates multi-omics feature data with prior information in the form of pathways for either regression or classification tasks. AKLIMATE uses a novel multiple-kernel learning framework where individual kernels capture the prediction propensities recorded in random forests, each built from a specific pathway gene set that integrates all omics data for its member genes. AKLIMATE has comparable or improved performance relative to state-of-the-art methods on diverse phenotype learning tasks, including predicting microsatellite instability in endometrial and colorectal cancer, survival in breast cancer, and cell line response to gene knockdowns. We show how AKLIMATE is able to connect feature data across data platforms through their common pathways to identify examples of several known and novel contributors of cancer and synthetic lethality.

## Author summary

We describe a new method that incorporates multimodal molecular measurements with gene pathway information for classification and regression prediction tasks. The method combines powerful machine learning methodologies such as ensemble, kernel and stacked learning. A key new contribution is the creation of empirical kernels from pairwise similarities of sample predictions and sample paths along the trees of a random forest specific to a particular gene module. We demonstrate the method performs as well as, or better than, top approaches on three very different cancer genomics applications.

This is a *PLOS Computational Biology* Methods paper.

## Introduction

The drop in sequencing cost has made it common for biological experiments to generate multi-omic profiles under a variety of conditions and perturbations. For example, the Cancer Genome Atlas (TCGA) contains thousands of patient samples with simultaneous copy number, mutation, methylation, mRNA, miRNA and protein measurements [1]. The analysis of multi-omic experiments produces feature sets that capture the genomic and transcriptomic changes relevant to a specific condition, sample subtype, or biological pathway—this “prior knowledge” eventually accumulates in a growing number of databases [2–6]. However, the interpretable integration of such prior knowledge with multi-omic data from new experiments remains an important challenge (see Materials and methods section for a discussion on challenges and previous approaches).

In this study, we introduce the Algorithm for Kernel Learning with Integrative Modules of Approximating Tree Ensembles (AKLIMATE)—a novel approach that combines heterogeneous data with prior knowledge in the form of gene sets. AKLIMATE can evaluate the predictive power of individual features (e.g. genes) as well as feature sets (e.g. pathways). It harnesses the advantages of Random Forests (RFs; native handling of continuous, categorical and count data, invariance to monotonic feature transformations, ease of feature importance computation), Multiple Kernel Learning (MKL; intuitive integration of overlapping feature sets), and stacked learning (improved accuracy) while avoiding many of their shortcomings. AKLIMATE relies on three major computational insights. First, it leverages biological knowledge and multiple data types by building a separate RF model for each distinct gene set (e.g. biological process, transcriptional signature, genomic location), with the ability to incorporate heavily overlapped feature groups without the undesirable side effects of other approaches (e.g. Group Lasso; see Materials and methods). Second, it uses co-classification patterns of sample pairs across decision trees within an RF model to compute a kernel similarity matrix (RF kernel) that is data driven yet capable of capturing complex non-linear feature relationships. Third, the RF kernel naturally re-weights the input features so that more informative ones make bigger contributions to kernel construction. This final property is key when dealing with feature sets in which some, but not all, of the features are informative for classification. We demonstrate that these insights lead to a general improvement in performance when AKLIMATE is compared against state-of-the-art algorithms on various classification and regression tasks.

## Results

We evaluated AKLIMATE on multiple prediction tasks—microsatellite instability in endometrial and colon cancer, survival in breast cancer, and small hairpin RNA (shRNA) knockdown viability in cancer cell lines. We benchmarked AKLIMATE against comparable methods that have performed well in recent Dialogue on Reverse Engineering and Assessment Methods (DREAM) challenges. We chose both classification and regression tasks as well as various levels of data availability—a single data type, multiple data types (including inferred data), or multiple data types with clinical information.

### Microsatellite instability

We first tested AKLIMATE on predicting microsatellite instability in the colon and rectum adenocarcinoma (COADREAD) and uterine corpus endometrial carcinoma (UCEC) TCGA cohorts. Microsatellite instability (MSI) arises as a result of defects in the mismatch repair machinery of the cell. Tumors with MSI (often accompanied by higher mutation rates) represent a clinically relevant disease subtype that is associated with better prognosis. MSI is also an immunotherapy indicator as such tumors produce more neoantigens. MSI can be predicted with high accuracy from expression alone [7], providing a straightforward benchmark for AKLIMATE performance with a single feature type in a binary classification setting.

We used expression data (upper-quantile normalized RSEM counts of RNA-Seq) and MSI annotations as described in [8] for the COADREAD and UCEC TCGA cohorts. The UCEC cohort consisted of 326 patients, of which 105 exhibit high microsatellite instability (MSI-H) and the remaining 221 are classified as either low (MSI-L) or stable (MSS). The COADREAD cohort included 261 samples, with 37 MSI-H and the remaining 224 classified as either MSI-L or MSS. In both tumor types, we trained models to distinguish MSI-H patients from MSI-L+MSS patients on 50 phenotype-stratified partitions of 75% training and 25% test folds. We then computed area under the ROC curve (AUROC) for each set of test fold predictions.

We compared AKLIMATE to Bayesian Multiple Kernel Learning (BMKL) because it performed well in several DREAM challenges, in particular winning the NCI-DREAM Drug Sensitivity Prediction Challenge [9]. Furthermore, its pathway-informed extension [7] shares several similarities with AKLIMATE—it is a multiple kernel learning method that operates on pathway-derived kernels. In particular, BMKL uses expression-based Gaussian kernels computed on features from the Pathway Interaction Database (PID) pathway collection [10]. We tested four versions of BMKL—sparse single-task BMKL(SBMKL), dense single-task BMKL(DBMKL), sparse multi-task BMKL (SBMTMKL), and dense multi-task BMKL (DBMTMKL). Sparse BMKL models are the focus of [7]—they use sparsity-inducing priors to train models with few non-zero kernel weights (we used the hyperparameters specified in [7]). We added DBMKL and DBMTMKL to the comparison because dense MKL models (almost all kernels receive non-zero weights) tend to produce higher predictive accuracy in many experimental settings (e.g. [11]). Their parameters were identical to the ones for the sparse models except for (*ζ*_*κ*_, *η*_*κ*_), which were set to (999, 1) in the dense models ((*ζ*_*κ*_, *η*_*κ*_) = (1, 999) in the sparse ones). Finally, the single-task models were trained separately on the UCEC and COADREAD cohorts, while the multitask versions learned MSI status on the two TCGA cohorts jointly, with each cohort representing a separate task. All train/test splits were matched across methods. All BMKL models used 196 PID gene sets; model parameters, kernel computations and data filtering steps matched the setup in [7].

Since AKLIMATE uses a much larger gene set compendium (*S* = 17, 273 feature sets, see S1 Text), we controlled for this prior information imbalance as a possible source of performance bias. For that purpose, we created a reduced version (AKLIMATE-reduced) that is restricted to the same set of 196 input PID sets as used by the original publication of BMKL. We refer to the full unrestricted model of AKLIMATE as simply AKLIMATE in this comparison.

**Fig 1:**
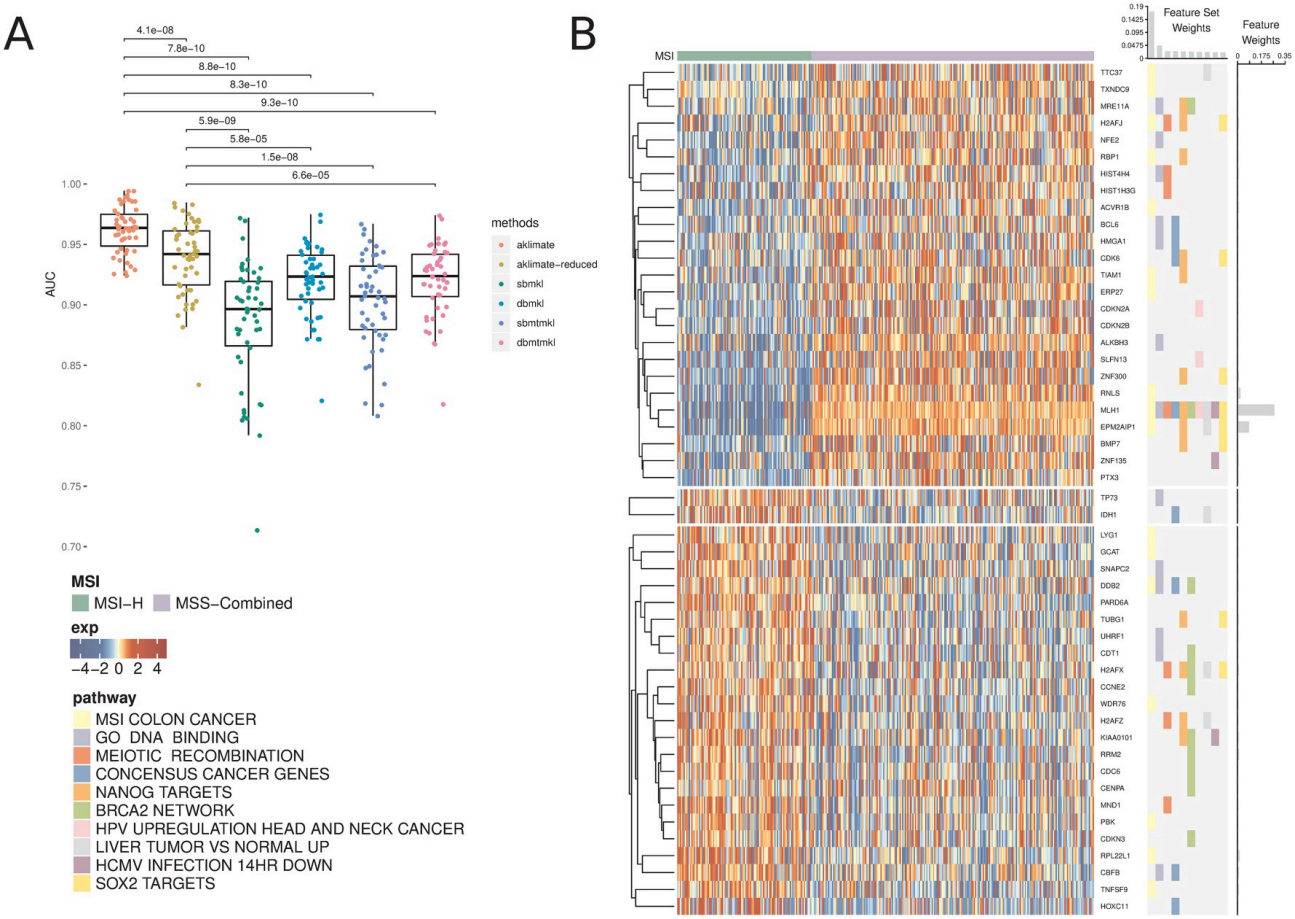
AKLIMATE performance on predicting MSI in UCEC TCGA. (A) Performance of AKLIMATE and BMKL on classifying MSI-H vs MSI-L+MSS. AUROC computed for 50 75%/25% stratified train/test splits. P-values for paired Wilcoxon signed rank test. Methods compared: aklimate—AKLIMATE on full collection of feature sets; aklimate-reduced—AKLIMATE with 196 PID pathways; sbmkl—sparse single-task BMKL; dbmkl—dense single-task BMKL; sbmtmkl—sparse multi-task BMKL; dbmtmkl—dense multi-task BMKL. Multi-task BMKL models are trained to simultaneously predict MSI status on UCEC and COADREAD cohorts. (B) Top 10 predictive AKLIMATE feature sets and top 50 predictive features. Expression of top 50 features (left heatmap); Membership of most predictive features in most predictive feature sets (right heatmap). Features are organized by KNN clustering into 3 groups, followed by hierarchical clustering within each cluster. Feature set model weights scaled to sum up to 1 (barplot, top of right heatmap). Feature model weights scaled to sum up to 1 (barplot, right of right heatmap). Feature and feature set weights averaged across 50 train/test splits.

AKLIMATE predicted MSI status in the UCEC cohort significantly better than AKLIMATE-reduced or any of the BMKL models (Fig 1A, mean AUROCs 0.885±0.051, 0.92±0.029, 0.903±0.039, 0.92±0.03 for SBMKL, DBMKL, SBMTMKL, and DBMTMKL respectively). In particular, AKLIMATE achieved mean AUROC of 0.962±0.019 compared to 0.938±0.031 for AKLIMATE-reduced (*P* < 4.1*e* − 08; paired Wilcoxon signed rank test), suggesting a predictive benefit to AKLIMATE’s larger collection of gene sets. A larger gene set collection is both more likely to contain sets derived specifically to describe the MSI process and more flexible in terms of the possible combinations of component gene sets. Indeed, the most informative feature set according to AKLIMATE was “MSI Colon Cancer” (16.9% relative contribution to model explanatory power)—a gene expression signature for MSI-H vs MSI-L+MSS in COADREAD cohorts [12] (Fig 1B). Futhermore, the next two most informative sets were “GO DNA Binding” (4.55% relative contribution) and “REACTOME Meiotic Recombination” (2.4% relative contribution), both of which are strongly relevant to DNA mismatch repair (MMR). Of note, the MutL Homolog 1 (MLH1) gene was the top-ranked single feature in AKLIMATE (26.7% relative contribution) and was present in the ten top ranked gene set kernels (Fig 1B). MLH1 is a key MMR gene involved in meiotic cross-over [13]—loss of MLH1 expression, usually through DNA methylation, is known to cause microsatellite instability. This demonstrates that AKLIMATE was able to pinpoint individual causal genes as it sifts through thousands of gene sets. In contrast, the meta-pathway constructed by AKLIMATE-reduced represents a poorer approximation to the underlying biological process, as evidenced by its lower AUROC. This is likely due to the fact that, out of the 196 PID pathways used, only “PID P53 Downstream” (35.2% relative contribution) contained MLH1 S2 Fig), limiting the influence of this key gene on the prediction task.

Interestingly, even though AKLIMATE-reduced used the same feature sets as BMKL, it achieved a statistically significant improvement over all BMKL varieties (Fig 1A). This included both the non-sparse and multi-task BMKL varieties, despite the fact that the latter drew benefit from an entire additional training set of COADREAD data. In this case, the difference in kernel representations may have contributed to the improved performance of AKLIMATE-reduced (see Discussion). For the COADREAD MSI classification task, we found that all methods performed equally well, with AKLIMATE-reduced having marginally lower accuracy compared to DBMKL & DBMTMKL and the full AKLIMATE model showing marginally higher mean AUROC compared to all BMKL varieties (S3 Fig). We suspect the strong MSI signal in the feature data makes this prediction task somewhat more facile, limiting our ability to discriminate among methods based on their predictive performance.

### Breast cancer survival

For our second benchmark, we considered the task of predicting survival in the Molecular Taxonomy of Breast Cancer International Consortium (METABRIC) cohort [14]. This problem is much more challenging than predicting MSI status, as demonstrated by the DREAM Breast Cancer Challenge [15] and elsewhere [16]. Another difference between this task and MSI inference is that the METABRIC cohort is annotated with curated clinical data. In fact, the clinical features are quite informative for survival prediction—pre-competition benchmarking by the DREAM Challenge organizers found that models that used exclusively clinical features significantly outperformed ones that used only genomic features, and performed only marginally worse than models in which clinical features were augmented by a subset of molecular features selected through prior domain-specific knowledge [17]. In addition, the best pre-competition clinical and molecular feature model had better accuracy than all but the top 5 models in the actual challenge [15].

A breast cancer model that foregoes the use of clinical data would clearly suffer from inferior performance as well as reduced relevance in real-world medical settings. To achieve clinical data integration, AKLIMATE introduces a special category of “global” features, which are added to the “local” features of each feature set prior to the construction of its corresponding Random Forest. Global features can therefore be interpreted as a uniform conditioning step applied to all AKLIMATE component Random Forests. In our METABRIC analysis, all clinical features are treated as global.

We compared AKLIMATE to two state-of-the art METABRIC survival predictors. The first one, which we refer to as the “Breast Cancer Challenge” (BCC) method, was the top-performer in the Sage Bionetworks–DREAM Breast Cancer Prognosis Challenge [15, 18]—an ensemble of Cox regression, gradient boosting regression, and K-nearest neighbors trained on different combinations of clinical variables and molecular-feature derived metagenes. The second one is a multiple kernel learning (MKL) method—Feature Selection MKL (FSMKL) [16]—that implements a pathway-informed extension of SimpleMKL [19] using linear and polynomial kernels created from clinical data and molecular features in pathways of the Kyoto Encyclopedia of Genes and Genomes (KEGG) [20]. In FSMKL, pathway features from different data types lead to the construction of separate kernels—in the case of METABRIC, each pathway produces distinct expression and copy number kernels (in contrast, AKLIMATE learns one kernel matrix from the combined pathway features across all data types). In addition, FSMKL treats each clinical feature as a singleton pathway that produces an individual kernel. To make our results directly comparable to FSMKL and BCC as presented in [16], we used a subset of the patient cohort (*N* = 639) and a reduced set of clinical variables to match the dataset used in that publication (see S1 Text).

We cast the problem as a classification task where molecular and clinical input features are used to predict whether a patient is alive or not at the 2000 day mark. Based on the 2000 day cutoff, there were 387 survivors and 252 non-survivors in the reduced cohort. Similar to the MSI analysis, we performed 50 stratified repeats of 80% train and 20% test partitions; AKLIMATE was trained on each training split and its accuracy computed on the respective test samples. To decrease computation time, AKLIMATE’s kernel construction step used 1000 trees instead of the default 2000 (see S1 Text for all other hyperparameter settings).

The full AKLIMATE model had higher mean accuracy than BCC (74.1±3.3% vs 73.3±0.2%) and was on par with FSMKL (74.1±3.3% vs 74.2±1.8%) with a slightly higher standard error across cross-validation folds compared with FSMKL (S4 Fig). The AKLIMATE accuracy was largely unchanged when it was restricted to use the same KEGG pathways as FSMKL (S5 Fig). All three algorithms selected clinical variables as important for prediction.

Of note, two of AKLIMATE’s main advantages were not fully utilized under these experimental settings. First, the benefit of incorporating prior knowledge is reduced because of the relatively low information content of genomic features. Second, the clinical variables may lack complex interactions, which may explain why modeling them with simpler linear techniques is just as effective. In fact, the most explanatory feature in the full AKLIMATE model (16% relative contribution) was the Nottingham Prognostic Index (NPI) [21]—a linear combination of tumor size, tumor grade, and number of lymph nodes involved. Even with these disadvantages, however, AKLIMATE achieved performance on par with two state-of-the-art methods that were specifically optimized for the METABRIC data.

**Fig 2:**
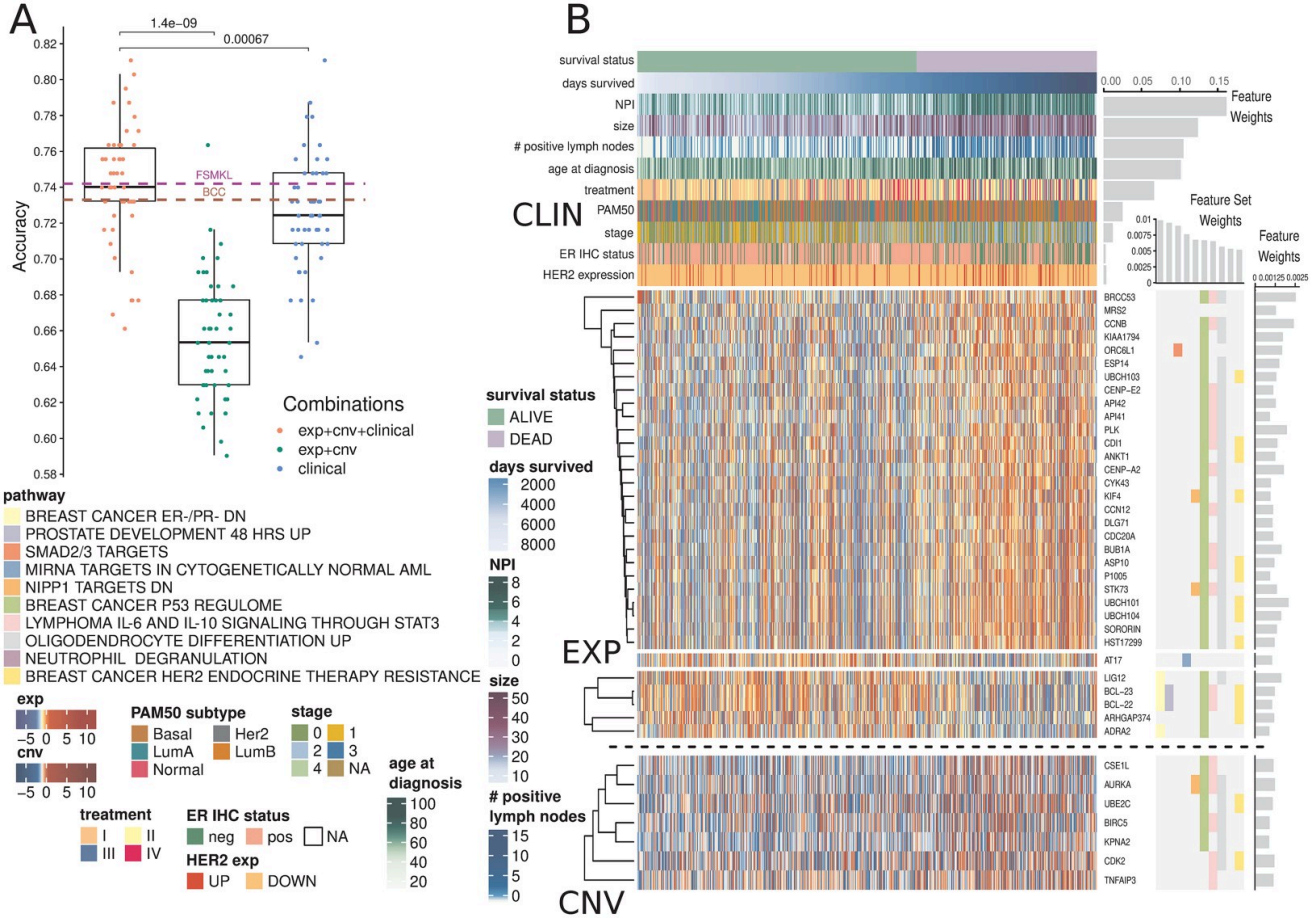
AKLIMATE performance on predicting survival at 2000 days in the METABRIC cohort. (A) Performance of AKLIMATE under different data type combinations. EXP+CNV—AKLIMATE with genomic features only; clinical—an RF model run with the clinical variables only; EXP+CNV+CLINICAL—AKLIMATE with genomic features as “local” variables and clinical features as “global” ones. FSMKL and BCC dashed lines show mean performances for the two models under 5-fold cross-validation as shown in [16]. (B) AKLIMATE results highlighting the top 10 predictive feature sets and top 50 predictive features. Figure organized as Fig 1. Clinical variables shown as column annotations; included only if among the 50 most informative features in the model. Clinical variables are ranked from top to bottom by relative predictive contribution. Survival status is a binary variable representing survival at 2000 days (labels) while days survived shows actual duration of survival. Samples sorted by days survived within the two classes. Feature and feature set weights averaged across 50 train/test splits.

In this task, clinical information proved to be the most influential data type for survival prediction. AKLIMATE models with clinical features alone were more accurate than AKLIMATE models with genomic features alone (p-val = 1.9e-08, paired Wilcoxon signed rank test, Fig 2A). This is even more striking considering the models used tens of thousands of genomic features versus only 15 clinical ones. However, the AKLIMATE models using both clinical and genomic features outperformed each single-component model (p-val = 1.4e-09 for full versus genomic, p-val = 6.7e-04 for full versus clinical, paired Wilcoxon signed rank test, Fig 2A), suggesting that the inclusion of genomic features contained complementary signals with respect to the clinical variables. The mean relative contributions of each data type in the full models were 61.6% for clinical, 29.6% for expression, and 8.8% for copy number.

Importantly, while the full AKLIMATE and FSMKL models achieved similar mean accuracy, the influence on prediction of individual features and pathways in each model were quite different. AKLIMATE heavily favored clinical variables, with 54.5% of the model’s relative explanatory power carried by just five features—NPI, tumor size, lymph node involvement, age and treatment (Fig 2B). FSMKL ranked tumor size as the most important clinical feature (second overall), with other clinical variables also generally considered relevant—e.g. NPI(9th), age (11th), histological type(14th), tumor group(34th) and PAM50(40th) [16]. Most clinical variables, however, were considered less informative than FSMKL’s top ranked KEGG pathway-based kernels. FSMKL’s highest weighted kernel was “Intestinal Immune Response for IgA production”, followed in importance by “Arachidonic acid metabolism”, “Systemic lupus erythematosus”, “Glycerophospholipid metabolism”, and “Homologous recombination”—all of these KEGG-based kernels scored higher than any clinical variable with the exception of tumor size [16].

AKLIMATE’s most informative feature sets were enriched for breast cancer progression and response to treatment, with 3 of the top 10 and 8 of the top 20 (S1 Table) related to these functional groups. For example, the most informative feature set (“BREAST CANCER ER-/PR- DN”) represents a signature that is correlated with reduced protein abundance of the estrogen (ER) and progesterone (PR) hormone receptors [22]. Similarly, the 9th most informative pathway (“BREAST CANCER HER2 ENDOCRINE THERAPY RESISTANCE”) [23] captures transcriptome changes associated with the development of resistance to targeted therapies. Both of these signatures serve as proxies for highly relevant information not available for the METABRIC cohort (protein activity for PR and therapy resistance). Furthermore, the latest American Joint Committee on Cancer breast cancer staging manual introduces tumor grade and ER/PR/HER2 receptor status among the key breast cancer biomarkers, which already include tumor size and lymph node engagement [24]. All of these appeared as highly informative AKLIMATE model features, either directly or via a proxy genomic signature.

Breast cancers are typically subtyped into one of several main pathological groups that heavily influence treatment selection (e.g. luminal / ER-positive, HER2-amplified, basal / triple-negative, normal-like). Appreciable differences in AKLIMATE’s METABRIC performance can be seen when the samples are divided up by their subtypes (based on PAM50 signatures [25], S6 Fig). The basals were associated with the least accurate (65.6% ± 8%, n = 131) and the luminal-As with the most accurate (80.8% ± 5.4%, n = 200) results, a trend that is expected given the known higher heterogeneity levels of less differentiated basal relative to luminal-A tumors.

### shRNA knockdown viability

We tested AKLIMATE’s ability to integrate multiple data types and solve regression tasks to predict cell viability post shRNA knockdown. In this case, the prediction labels were continuous values representing cell line survival after the shRNA-mediated mRNA degradation of a particular gene. Gene profiles were computed with the Analytic Technique for Assessment of RNAi by Similarity (ATARiS) method [26] that creates consensus scores by combining viability phenotypes from multiple shRNAs targeting the same gene. We selected 37 such profiles for different genes across 216 cancer cell lines from the Cancer Cell Line Encyclopedia (CCLE) [27]. We chose these 37 tasks (out of 5711 available consensus profiles) because they had at least 10 cell lines showing strong (>2 standard deviations from mean) knockdown viability response and were in the top quartile by variance of all consensus profiles (see S1 Text). Based on the results of the DREAM9 gene essentiality prediction challenge [28], we expected this task to be more difficult than the other case studies.

We used expression and copy number measurements from CCLE [29] as predictive features. We augmented these two data types by adding discrete gene copy number calls made by GISTIC2 [30] and activities for 447 transcriptional and post-transcriptional regulators inferred by hierarchical VIPER [31, 32]. We focused on the 206 cell lines for which we had knockdown profiles, copy number and expression features.

We compared AKLIMATE’s performance to three of the five top performing methods in DREAM9 [28] as well as three baseline algorithms. We briefly describe the DREAM9 subchallenge 1 top performers next. Multiple Pathway Learning (MPL) [31] took 5th place (see https://www.synapse.org/#!Synapse:syn2384331/wiki/64760 for challenge results)—it used elastic-net regularized Multiple Kernel Learning with Gaussian kernels based on feature sets from the Molecular Signature Database (MSIGDB) [3]. MPL and Random Forest Ensemble (MPL-RF) took 2nd place—its prediction was based on averaging a Random Forest classifier with MPL. MPL and MPL-RF were our contributions to the DREAM9 challenge. Both methods were run with the same hyperparameters and pathway collections as in DREAM9 [28, 31]. Kernelized Gaussian Process Regression (GPR) took 3rd place—it used extensive filtering steps to reduce the input feature dimensionality, followed by principal component analysis, and finally Gaussian Process regression with covariance computed from the principal components [28] (see also https://www.synapse.org/#!Synapse:syn2664852/wiki/68499 for implementation and model description). We downloaded the code from the Synapse URL and ran it with the published DREAM9 hyperparameters.

To provide performance baselines, the DREAM9 winners were augmented by standalone Random Forest (RF), Generalized Linear Model (GLM) with lasso penalty (GLM-sparse), and GLM with L2 regularization (GLM-dense). RF was run with the *ranger* R package [33] with the following hyperparameters—sampling without replacement with 70% of the samples used for tree construction, minimum node size of 10, 1500 trees and 10% of the features randomly sampled for each node split. GLM-dense and GLM-sparse were run using the *glmnet* R package [34] with the response family set to “gaussian” and the strength of regularization *λ* optimized through cross-validation. The elastic net tradeoff between the lasso and ridge penalties *α* was set to *α* = 0.8 (GLM-sparse) and *α* = 0.001 (GLM-dense).

We did not include the nominal first place winner of DREAM9—an ensemble of four kernel ridge regression models with kernels trained through Kernel Canonical Correlation Analysis and Kernel Target Alignment—because we could not re-run the source code supplied with the challenge submission. We feel this omission is not material as the top 3 methods were declared joint co-winners—their results were shown to be statistically indistinguishable from each other but separable from the rest of the entries [28]. Furthermore, experiments in [31] suggest that this method underperforms MPL, MPL-RF and GPR when only high-quality shRNA knockdown profiles are considered.

To save computational time, we compared methods on a single stratified train/test split for each ATARiS profile (a different split for each profile) where 67% of the cell lines were used for training and 33% were withheld for testing. Each method was run with its recommended parameters and filtering steps—if no filtering steps were specified, the method used all available features. AKLIMATE’s prediction binarization quantile was set to its default value of *q* = 0.05 (see Methods, also S1 Text for all other settings).

**Fig 3:**
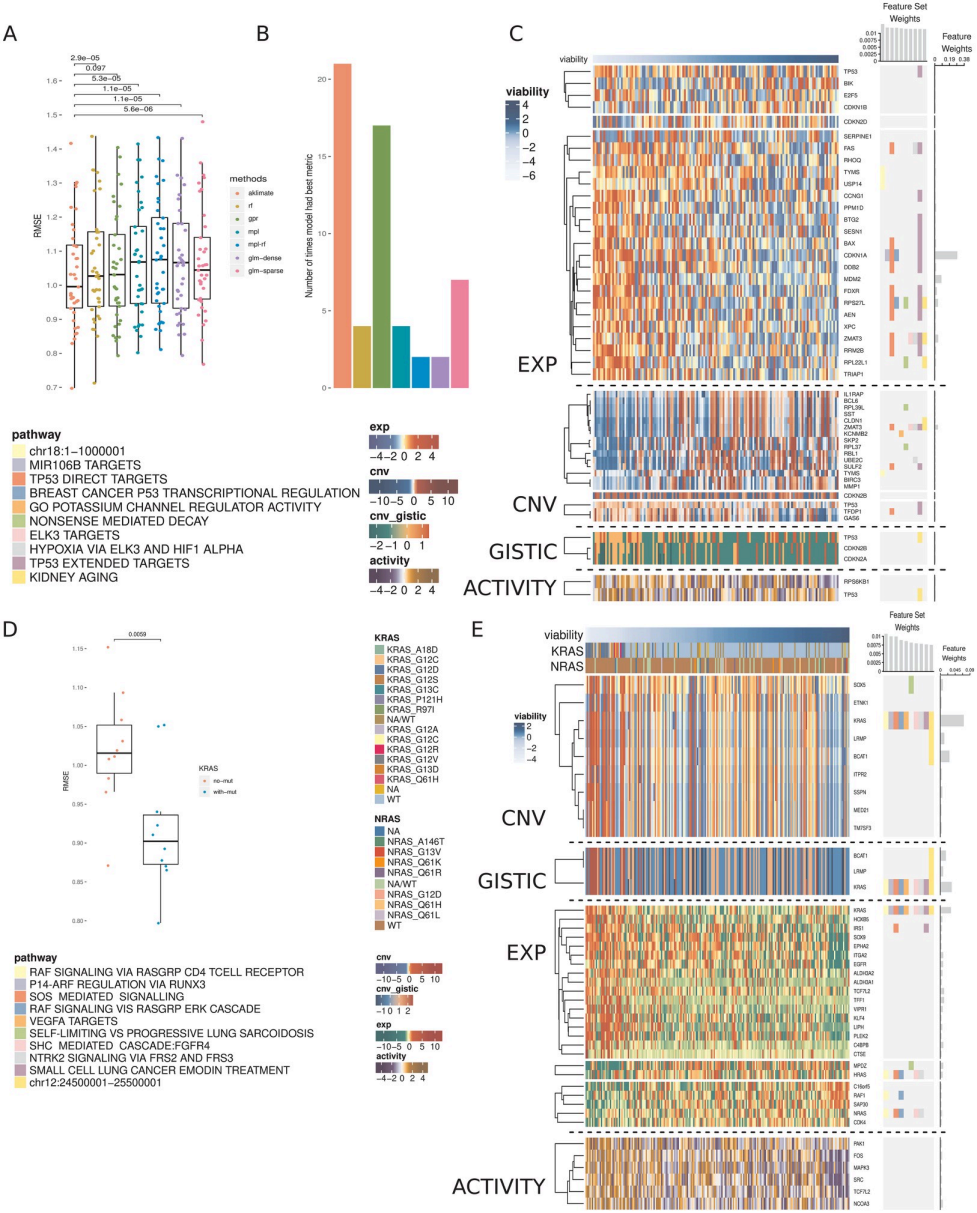
Prediction of cell line viability after shRNA gene knockdowns. (A) RMSEs of AKLIMATE and competing methods on 37 consensus viability profiles from the Achilles dataset. P-values for RMSE comparisons from paired Wilcoxon signed rank test. Methods: Random Forest (RF), Gaussian Process Regression (GPR), Multiple Pathway Learning (MPL), ensemble of MPL and Random Forest (MPL-RF), L2 regularized linear regression (GLM-dense), L1 regularized linear regression (GLM-sparse). (B) Number of times an algorithm produced the best RMSE on a prediction task. To prevent small relative RMSE differences from having a biasing effect on the win counts, for each task we consider all algorithms with RMSE within 1% of the min RMSE to be joint winners. For that reason, the total win counts add up to more than the number of regression tasks. (C) AKLIMATE top 10 informative feature sets and top 50 informative features for the task of predicting MDM4 shRNA knockdown viability. Organized as Fig 1. (D) RMSEs of KRAS AKLIMATE models with and without the use of mutation profiles for eight key regulators. Results shown for 10 matched stratified train/test splits where 80% of the cohort is used for training and 20% for testing. (E) AKLIMATE top 10 informative feature sets and top 50 informative features for the task of predicting KRAS shRNA knockdown viability when mutation features are used (feature and feature set weights averaged over 10 train/test splits). Organized as Fig 1.

AKLIMATE achieved average Root Mean Squared Error (RMSE) of 1.031 vs 1.048 for GPR, 1.056 for RF, 1.066 for GLM-dense, 1.07 for MPL, 1.071 for GLM-sparse and 1.081 for MPL-RF. While the improvements were modest, the mean RMSE difference was statistically significant in all but one case (Fig 3A). AKLIMATE was also the top performer when we considered the number of times an algorithm achieved the best RMSE on an individual prediction task (Fig 3B, AKLIMATE retained top spot under non-RMSE metrics as well—S7 Fig). AKLIMATE performed better than average across nearly all tasks (S8 Fig); its advantage was particularly pronounced in predicting the essentiality of key regulators—Beta-Catenin (CTNNB1), Forkead Box A1 (FOXA1), Mouse Double Minute 4 Homolog Regulator of P53 (MDM4), Phosphatidylinositol-4,5-Bisphosphate 3-Kinase Catalytic Subunit Alpha (PIK3CA)—or housekeeping genes—Proteasome 26S Subunit ATPase 2 (PSMC2), and the fifth ATPase subunit (PSMC5). As these gene classes are heavily studied and thus over-represented in our pathway compendium, AKLIMATE’s enhanced accuracy may be due to the relatively higher abundance of relevant prior knowledge.

For example, AKLIMATE’s ability to predict MDM4 knockdowns benefited from MDM4’s function as a p53 inhibitor—many of the most informative feature sets in the MDM4 model relate to p53’s regulome and its role in controlling apoptosis, hypoxia and DNA damage response (Fig 3C). The top features were also functionally linked to p53—Cyclin Dependent Kinase Inhibitor 1A (CDKN1A, 28.6% relative contribution) is a kinase that controls G1 cell cycle progession and is tightly regulated by p53; Mouse Double Minute 2 homolog P53 binding protein (MDM2, 8% relative contribution) participates in a regulatory feedback loop with p53; Zinc Finger Matrin-Type 3 (ZMAT3, 3.7% relative contribution of expression; 3.5% of copy number) interacts with p53 to control cell growth. In addition, the four p53 features from each data type were all present among the 50 most informative ones. Individually they carried little signal (relative contribution: 0.5% expression, 0.3% copy number, 0.3% inferred protein activity, 0.2% GISTIC score) but taken together they clearly implicated p53 as one of the top 10 most informative genes. The ability to capture such multi-omic interactions is one of AKLIMATE’s main strengths, made possible by the use of RF kernels that fully integrate all data types. The synergy between different p53-based features is impossible to observe in methods that assign individual kernels to each data type (e.g. BMKL, FSMKL and MPL). Encouragingly, AKLIMATE dominated MPL/MPL-RF on almost all tasks even though they share the same MKL solver (Figs 3A and S8 Fig).

FOXA1 knockdown offered another example of AKLIMATE showing superior predictive performance on a well-studied gene. FOXA1 dysregulation is an essential event in breast cancer progression and subtype characterization. Due to breast cancer’s prevalence and clinical importance, there is an extensive list of relevant signatures in our feature set compendium. Eight of the top 10 (and 12 of 14 overall) feature sets in the FOXA1 AKLIMATE model were directly related to breast cancer experiments under different conditions (S10 Fig). As expected, FOXA1 features were the most informative (relative contribution: 31.5% FOXA1 expression; 3.4% FOXA1 inferred activity), with the androgen receptor (AR) also among the top 5 most informative genes (relative contribution: 2.8% AR expression).

Out of the 37 shRNA prediction tasks we considered, shRNA knockdown of the Kirsten Rat Sarcoma Viral Oncogene Homolog (KRAS) was the most obvious example of a task involving a well-characterized gene that did not experience discernible accuracy improvement over competing methods (S8 Fig). Our hypothesis is that a true biological “driver” is absent from the set of molecular features presented to AKLIMATE. To test this, we added a mutation data type containing information for 8 key regulators (KRAS, NRAS, PIK3CA, BRAF, PTEN, APC, CTNNB1 and EGFR), 3 of which (KRAS, CTNNB1, PIK3CA) have knockdown profiles among the 37 shRNA prediction tasks. Even with the addition of these limited 8 features we observed dramatic improvement in KRAS shRNA prediction accuracy across all metrics (Fig 3D, mean RMSE 0.918±0.025 vs 1.02±0.024; mean Pearson 0.665±0.023 vs 0.529±0.025; mean Spearman 0.57±0.039 vs 0.453±0.034). Furthermore, the KRAS mutation feature was by far the most informative (23.5% relative contribution, Fig 3E), followed by KRAS copy number (6.9%), KRAS GISTIC (3.3%) and KRAS expression (3.1%). While KRAS expression, copy number and GISTIC features all appeared among the 50 most informative ones in the “no mutation” run, their combined relative contribution was only 3.33% (1.5% copy number, 1% expression, 0.8% GISTIC, S11 Fig). Thus, the addition of the KRAS mutation feature was not only key to improving the predictive performance of the model on its own, but it also helped “lift” the influence of KRAS features form other data types.

Our training features are measured at the gene level, but AKLIMATE has no constraints on the inclusion of more granular data. For example, it can evaluate the importance of mutations at particular amino acid positions or the type of resulting substitutions. In the case of KRAS, glycine replacement by either aspartic acid or valine in the 12th amino acid position appears to have the biggest negative effect on cell viability post-shRNA knockdown (Fig 3E). G12 is a well-known KRAS mutation hotspot [35]—AKLIMATE’s ability to prioritize relevant hotspots can be a key advantage in modeling drug response or recommending treatment strategies.

The addition of mutation data did not yield any predictive benefit in modeling PIK3CA or CTNNB1 post-knockdown viability (S12 and S13 Figs). CTNNB1 had only nine mutations in the cohort, all of them in different codons. PIK3CA had 30 mutations, with some hotspots—the lack of improvement in this case may be due to the change in protein sequence not being biologically relevant, the ability of other genomic features to fully capture the mutation signal, or the fact that the “no mutation” models were quite accurate to begin with.

## Discussion

Recent surveys of cancer genome landscapes have shown that alterations of a particular pathway can involve many different genes and many different kinds of disruptions—for example, RB1 mutation, RB1 methylation, or CDKN2A deletion can all lead to aberrant cell proliferation [36]. Consequently, many bioinformatics approaches seek to combine data at the level of a biological process to benefit machine-learning applications in the cancer genomics setting. However, data platform diversity often prohibits such integration—variables can be of different scales (e.g. copy number vs gene expression) or different types (continuous DNA methylation, binary mutation calls, ordinal inferred copy number estimates). AKLIMATE’s early integration approach is a potential solution to capturing complementary, and possibly highly non-linear, information spread across data modalities—all data types are considered, and potentially used, when supervised training constructs a Random Forest-based kernel for genes that function together in a common cellular process. In contrast, MPL, FSMKL and other MKL approaches that employ unsupervised kernel construction compute segregated data-type specific kernels for each pathway and let the linear combination “meta-kernel” determine their optimal combination. This may result in sub-optimal solutions—features that belong to the same pathway but are in different data types can now only interact with each other on the kernel level and not individually. AKLIMATE’s approach creates a richer interaction model that is flexible enough to capture same-gene, cross-gene, and cross-data type interactions.

Another limitation of current pathway-informed kernel learning methods is that a single informative feature can go undetected if only present in large pathways—if all member features contribute equally to kernel construction, the importance of the relevant feature is obscured by the non-relevant majority. In contrast, AKLIMATE’s RF kernels effectively allow individual features to influence the model. This advantage is illustrated by the improvement in AKLIMATE performance over BMKL on the MSI prediction task. BMKL’s Gaussian kernels treat each feature in a feature set as equally important in the computation of the respective kernel matrix. As MLH1 appears only once in the PID pathway compendium, its contribution is masked by less informative features. On the other hand, due to the supervised manner of their construction, AKLIMATE’s RF kernels inherit an RF’s ability to prioritize features based on their relevance to the classification task—informative features are by definition overrepresented among tree node splitting variables. As all components of the RF kernel are derived from properties of the RF trees, informative features exercise proportionately higher influence over the RF kernel construction. Therefore, if only a small subset of a pathway’s features are truly relevant, they can be clearly distinguished from (thousands of) non-relevant ones. For example, both AKLIMATE and AKLIMATE-reduced pick MLH1 as the most informative feature (26.7% and 16.4% relative contribution respectively), with a steep decline in the importance of the next best feature (AKLIMATE—EPM2AIP1, 8% relative contribution; AKLIMATE-reduced—PARD6A, 4.8% relative contribution).

In addition to filtering out non-relevant genes from a gene set definition, an AKLIMATE model can also facilitate the repurposing of seemingly unrelated prior knowledge—e.g. a gene set collected from one study is predictive in another one where the connection between the two studies is not obvious. For example, in the UCEC MSI prediction task, AKLIMATE selected a gene set that is upregulated upon HPV infection in head-and-neck cancer. The connection between HPV infection (which knocks out TP53) and UCEC microsatellite instability involves the DNA damage and repair machinery. In this case, it makes sense that a gene set associated with DNA repair would be selected as a predictive feature of MSI status (even for a different tumor type). In other cases, the relationship between an informative feature and the prediction task itself may not be as obvious. As collections of gene sets continue to grow, especially due to contributions from less curated sources like the results of high-throughput studies (e.g. gene clusters from an RNA-Seq experiment), the number of gene combinations that could serve as predictive biomarkers increases—leveraging this form of prior knowledge will thus continue to grow in importance.

A further key advantage of AKLIMATE is its ability to accommodate variables that do not readily map to genome-based feature sets (e.g. clinical data). In cases such as METABRIC, where clinical features provide much of the predictive power, the most salient question is how to identify orthogonal genomic features conditioned on a set of given clinical data. Posing the problem this way reflects the practical situation in a hospital setting where clinical information is nearly always on-hand to treating physicians. AKLIMATE and FSMKL illustrate two different ways of incorporating such information. FSMKL treats each clinical variable as a feature set of size one and constructs a kernel for each of them individually. This approach is viable, but quite restrictive in how it models clinical variable interactions. While AKLIMATE can accommodate such a setup, it also permits a more complex representation of the way features interact—each clinical feature is of a special “global” type that gets included in every feature set. The “global”-“local” feature hierarchy allows flexibility in modeling interactions among clinical variables and between clinical variables and genomic features. Such a hierarchy is necessary when features work on different biological scales—for example, tumor grade is an organ-level characteristic that captures a snapshot of the behavior of millions of cells and is therefore likely to have a different information content compared to the copy number status of an individual gene.

An important aspect of AKLIMATE’s use of prior knowledge is its ability to identify relevant features even in cases where many confounders exhibit high collinearity. This problem is similar to the one encountered in genome-wide association studies where an allele conferring a phenotype of interest can exist in a large haplotype block containing the alleles of many other irrelevant “hitchhiking” genes. In such situations, prior knowledge often helps researchers select the true causal variant among potentially many false positives. Consider AKLIMATE’s top two most informative features for the MSI prediction task—MLH1 and EPM2AIP1. EPM2AIP1 is on the DNA strand opposite to MLH1, shares a bi-directional CpG island promoter with it and can be concurrently transcribed [37]. The transcriptional profiles of the two genes are nearly identical (Fig 1B)—in the absence of other information it would be extremely difficult to prioritize the “driver” (MLH1) over the “passenger” (EPM2AIP1) using expression data alone. AKLIMATE’s feature sets provide the necessary prior knowledge—while EPM2AIP1 is indeed deemed the second most informative feature, its relative contribution is over three times smaller than that of MLH1.

AKLIMATE’s robustness to false positives is not limited to expression features—it can prioritize relevant genes even if they are subject to large-scale copy number events and thus have almost identical copy number profiles with many other genes. We observed this effect in the MDM4 knockdown prediction task (Fig 3C) as well as KRAS knockdown prediction with mutation data (Fig 3E). In the former, there is a clear large scale copy number event that involves 7 of the 50 most predictive genes, but ZMAT3 is given by far the highest weight because of the biological prior of the feature sets. Similarly, in the latter 9 copy number features have very similar profiles, but the KRAS one is prioritized as the most important. The KRAS GISTIC feature is also favored among a group of genes affected by large-scale events—an indicator that the robustness to collinearity extends beyond continuous to categorical features.

Making use of prior knowledge, such as the results of past experiments, is often a key component in the success of machine-learning applications in genomics analysis [17, 38]. AKLIMATE uses a biologically-motivated prior distribution on the feature space—as demonstrated, this approach often outperforms methods that use a uniform prior over input features. AKLIMATE updates its prior information in a data driven manner—therefore, the feature set compendium does not have to be tailored to the problem at hand, avoiding the need for problem-specific filtering heuristics. When high quality relevant prior experiments are available, AKLIMATE tends to perform better. For example, the most important feature set for MSI prediction in endometrial cancer is a previously published signature characterizing MSI in colon cancer (Fig 1B). From that perspective, AKLIMATE acts as a framework for prioritizing past experiments that are most relevant to the interpretation of a new dataset.

By aggregating feature weights within individual data types, AKLIMATE can also be used to rank data type contributions. For example, while expression is generally most important in predicting shRNA knockdowns (mean importance 71.6% across tasks), there are cases where copy number is more informative, such as PSMC2(61.7%), RPAP1(55%) and CASP8AP2(47%) (S9 Fig). Furthermore, inferred protein activity varies tremendously in terms of its contribution—from < 1% in predicting PSMC2 knockdowns to 27.7% for STRN4 (S9 Fig). AKLIMATE’s ability to zero in on information-rich data types could help in designing targeted future experiments.

From a stacked learning perspective, AKLIMATE augments standard level-one cross validated predictions with topological aspects of the base RFs—how often data points end up in the same leaf node and whether they tend to end up in early-split or late-split leaves. Such augmentation improves performance—in all the cases we studied, AKLIMATE outperforms both the best individual base learner and the ensemble that averages base learner predictions (S1 Fig). In addition, AKLIMATE does better than a standard Super Learner (regularized linear regression meta-learner) although without achieving statistical significance on some of the data sets (S1 Fig). This suggests that propagating additional information from a base learner (beyond the predictions it makes) into the level-one data can lead to a more accurate meta-learner. While AKLIMATE provides a blueprint for tree-based algorithms, using other base learners might yield better results.

AKLIMATE requires minimal feature pre-processing, can query tens of thousands of feature sets and its main steps are trivially parallelizable. It performs as well as, or better than, state-of-the-art algorithms in a variety of prediction tasks. AKLIMATE can natively handle continuous, binary, categorical, ordinal and count data, and its feature sets are easily extendable. For example, gene expression data can be augmented by epigenetic measurements (e.g. DNA methylation or ATAC-Seq), mutations in promoters or enhancers, splice variant proportions, and so on. Furthermore, slight modifications to AKLIMATE could incorporate prior importance scores for features within a feature set as well as structured relationships such as known feature-feature interactions (e.g. transcription factor to target gene). To simplify the exposition, we have focused our derivations and applications to regression and binary classification problems. However, AKLIMATE is readily extendable to the multi-class setting and the current public code base provides such an implementation.

While AKLIMATE demonstrates enhanced predictive power, future improvements are certainly possible that could lead to a bigger performance boost. For example, a kernel that captures the topological distance between samples in RF trees as suggested in [39] could provide a more accurate replacement for the *K*_2_ kernel we introduced. Sample-weighting schemes during kernel construction could be investigated as well. For example, [40] suggests a weighted RF kernel where sample contributions depend on the predictive accuracy of their assigned leaves or the classification error rate for an individual sample.

Finally, even though we present examples drawn from the field of bioinformatics, AKLIMATE can be applied to any task with multi-modal data and prior knowledge in the form of feature groups, particularly when the feature groups have evidence that span data types.

## Materials and methods

### Background

Before describing AKLIMATE in detail, we briefly summarize existing approaches to which AKLIMATE’s design and performance can be compared. There are three main challenges inherent to bioinformatics algorithms for supervised and unsupervised learning—prior knowledge integration, heterogeneous data interrogation, and ease of interpretation. New methods try to address some aspects of these three challenges [41–43]. Several common approaches emerge for supervised learning tasks. A popular way of increasing interpretability is to train models with regularization terms that constrain the number of included features—sparse models are considered easier to interpret than ones containing thousands of variables. Common regularization penalties are the lasso [44] and the elastic net [45]. More sophisticated regularization schemes can control the model behavior at the feature set level (e.g. the group lasso (GL) [46] and the overlap group lasso (OGL) [47]), allowing the incorporation of prior knowledge in the form of feature sets. However, both have drawbacks that make them less suitable for biological analysis where genes often participate in multiple processes. GL requires that if a feature’s weight is zero in one group, its coefficients in all other groups must necessarily be zero. OGL tends to assign positive coefficients to entire feature sets, making it less suitable in situations where only some of a set’s features are relevant.

Network-regularized methods [48, 49] represent a different approach where gene-gene interaction networks are used as regularization terms on the L_2_-norm of feature weights. While they do use pathway-level information and are straightforward to interpret, they generally focus on an individual data type.

Multiple Kernel Learning (MKL) approaches [7, 16, 19, 50–52] can incorporate heterogeneous data by mapping each set of features through a kernel function and learning a linear combination of the kernel representations. Each kernel represents distinct sample-sample similarities providing flexible and powerful transformations to access either explicit or implicit feature combinations. To prevent overfitting, MKL methods generally include a regularization term—e.g., L_1_ sparsity-inducing norm on the kernel weights [19] or the elastic net [51]. Prior knowledge can also be integrated by constructing individual kernels from a pathway’s member features within each data type [7, 16, 28, 31, 52]. Indeed, MKL methods with prior knowledge integration [9, 28] have won several Dialogue on Reverse-Engineering Assessment and Methods (DREAM) [53] challenges, including a predecessor of the approach described here [28, 31]. Nevertheless, MKL suffers drawbacks when the contributions of individual input features need to be evaluated. Except in trivial cases, it is generally impossible to assign importance to the original features once the method is trained in the kernel function feature space, thus limiting interpretability of solutions. In addition, feature heterogeneity necessitates the construction of separate kernels for each data type, limiting the ability of MKL to capture cross-data-type interactions.

All of these methods can be used as components in more complex ensemble learning models. Ensembles combine predictions from multiple algorithms into a more robust, and often more accurate, “wisdom of crowds” final prediction. The simplest and most common ensemble technique is averaging the predictions of component models. Averaging over uncorrelated models or models with complementary information can improve performance—it is one of the main reasons for the emergence of collaborative competitions such as the DREAM challenges [9, 54]. In fact, such ensemble methods often win DREAM challenges [18] or outperform competitors in genomic prediction tasks [55]. While they provide a boost in predictive accuracy, their interpretation is quite challenging—ensembles often combine different model types, making the computation of input feature importance impossible in the large majority of cases. A notable exception is the Random Forest (RF) [56]—an ensemble of decision trees that has been widely applied to bioinformatics problems.

A more general approach to combining the predictions of multiple models is model stacking [57]. In stacking, component (base) learner predictions are used as inputs to an overall (stacked) model which produces the final calls. To reduce overfitting and improve generalization, base learner predictions are generated in a cross-validation manner—a sample’s predicted label comes from a model trained on all folds except the one that includes the sample in question. Importantly, if the stacked model is a weighted combination of the base models (a Super Learner), it is asymptotically guaranteed to perform at least as well as the best base learner or any conical combination of the base learners [58, 59]. Stacked models exhibit identical pros and cons as standard ensemble models, with diminished interpretability traded off for increased accuracy. In some cases, however, interpretability can be tractable—e.g. [60] uses RF base learners with a stacked least square regression to compute a final weighted average of the base RF predictions. Although not examined in [60], we demonstrate that a similar setup can lead to an intuitive derivation of feature importance scores.

### Overview

**Fig 4:**
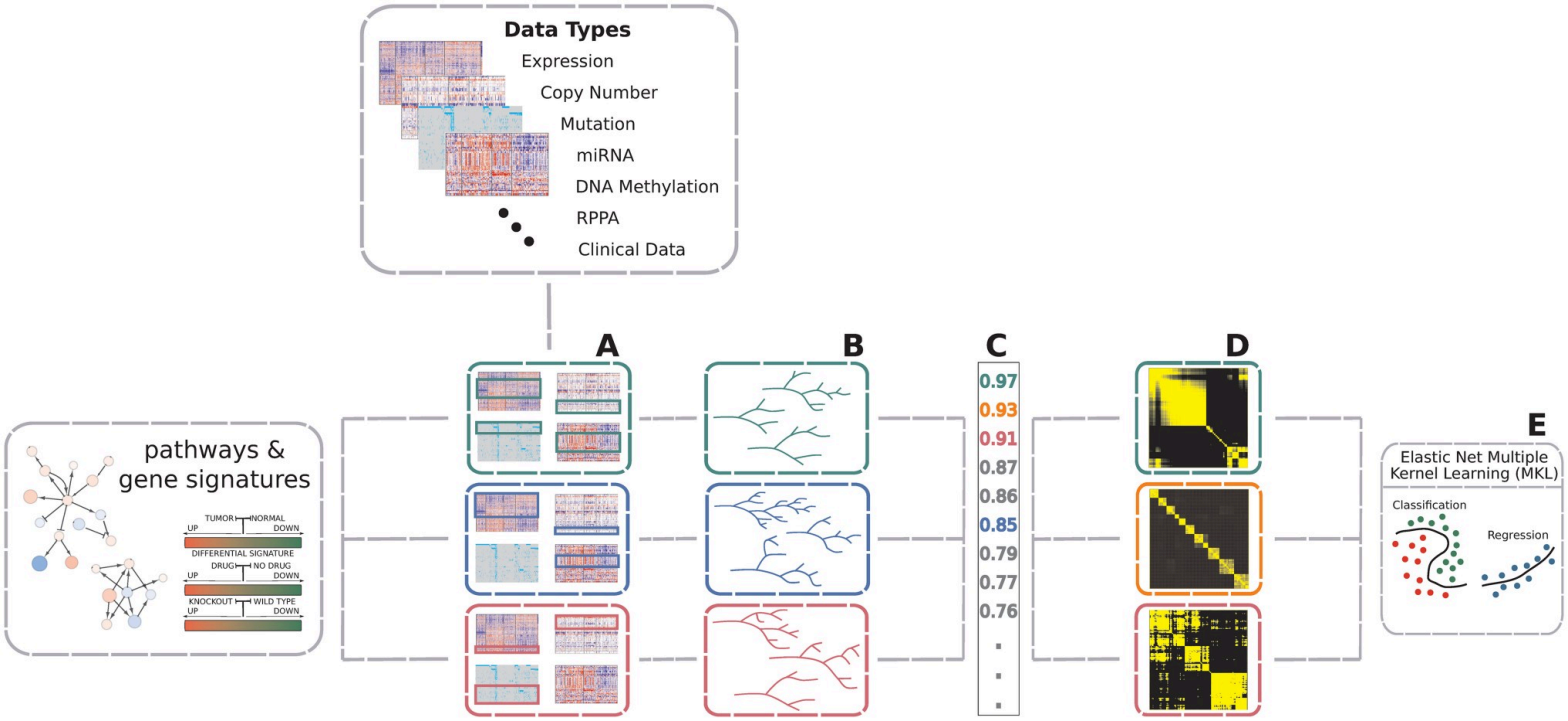
Overview of AKLIMATE. AKLIMATE takes as inputs multiple data types and a collection of feature sets. (A) Each feature set is overlapped with the available data types to identify all features that map to it (some feature sets might not extract features from all data types). (B) A feature-set-specific Random Forest model is trained on the multi-modal data identified in A. (C) RF models are ranked based on their predictive performance (metrics for mock RF models in A have matching color). (D) The top ranked RF models are converted to RF kernels (mock kernels shown for RF models with top 3 performance scores). (E) An elastic net MKL meta-learner finds the optimal combination of RF kernels and computes the final predictions. Elastic net hyperparameters are optimized via cross-validation.

**Fig 5:**
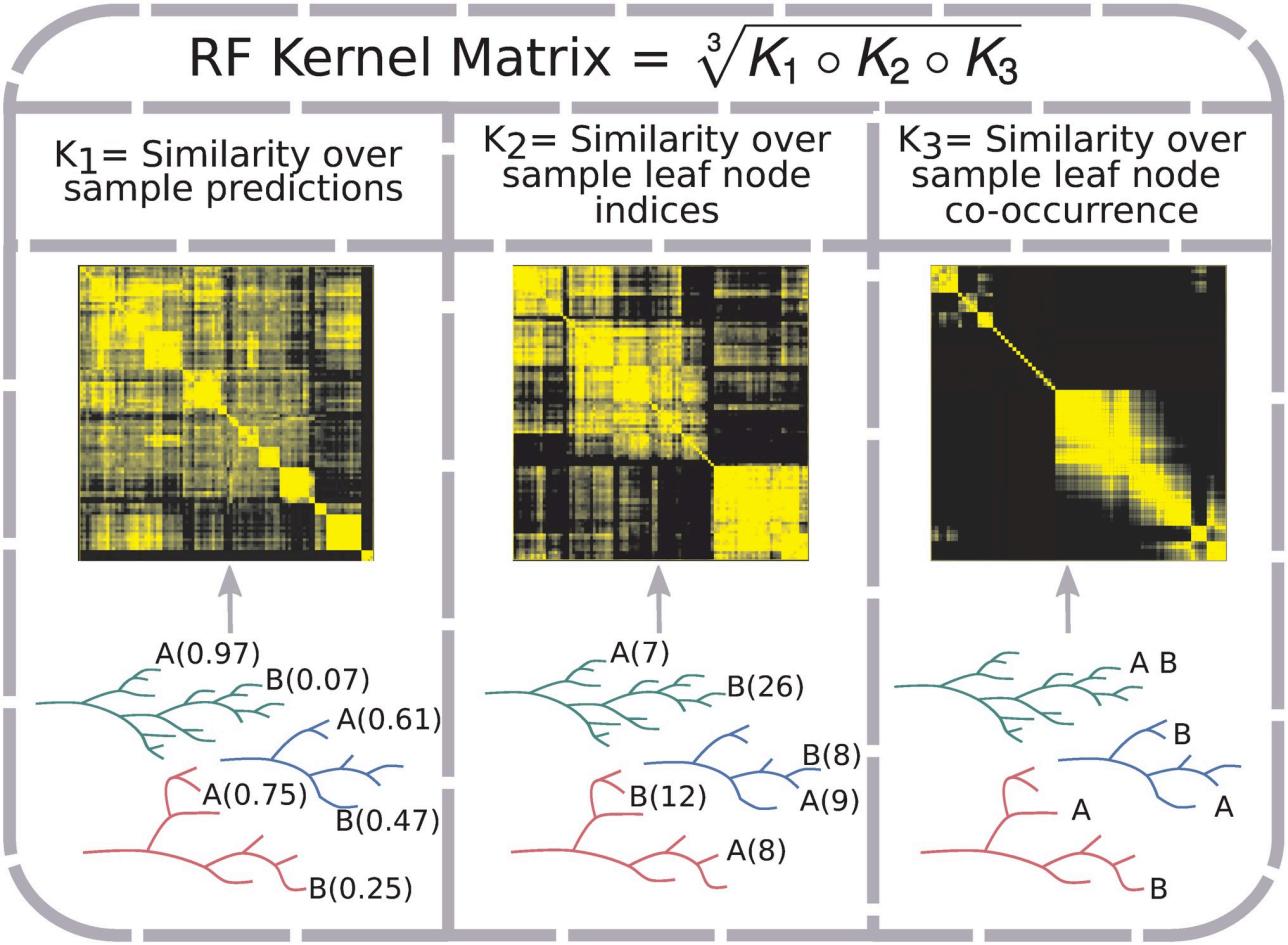
RF kernel matrix construction. The RF Kernel matrix is a geometric mean of the Hadamard product of three component similarity matrices. Each component captures a different aspect of a RF model. *K*_1_ captures the similarity over RF tree predictions for sample labels (in the case of classification, probability of belonging to a class); two samples (A and B) show different predicted probabilities of belonging to the positive class (probability estimate in parentheses). *K*_2_ represents the similarity over RF tree leaf indices to which samples are assigned; predicted leaf indices shown in parentheses for the two samples. *K*_3_ reflects the proportion of times samples are assigned to the same RF tree leaf; e.g. the two samples end up together in one out of three example trees.

AKLIMATE is a stacked learning algorithm with RF base learners and an MKL meta-learner. Each base learner is trained using only the features from a pre-defined feature set representing a known biological concept or process—e.g. a biological pathway, a chromosomal region, a drug response or shRNA knockdown signature, or disease subtype biomarkers (Fig 4). Each feature in a feature set can be associated with multiple data modalities—i.e. a gene will have individual features corresponding to its copy number, mutation status, mRNA expression level, or protein abundance. Furthermore, although feature sets normally consist of genes, they are easily extendable to a more comprehensive membership. For example, they can be augmented with features for mutation hotspots, different splice forms, or relevant miRNA or methylation measurements. AKLIMATE’s MKL meta-learner trains on RF kernel matrices (see the *RF Kernel* section), each of which is derived from a corresponding base learner (Fig 5). An RF kernel captures a proximity measure between training samples based on the similarity of their predicted labels and decision paths in the trees of the RF. The MKL learning step finds the optimal weighted combination of the RF kernels. The MKL optimal solution can be interpreted as the meta-kernel associated with the most predictive meta-feature set derived from all interrogated feature sets.

The key contributions of AKLIMATE are

1. The introduction of an integrative empirical kernel function that combines similarity in predicted labels with proximity in the space of RF trees (RF kernel).
2. The extension of kernel learning to a stacking framework. To our knowledge, AKLIMATE is the first stacked learning formulation to incorporate base kernels.

In the next sections we give a detailed description of each AKLIMATE component.

### Random forest

An RF learner is an ensemble of decision trees each of which operates on a perturbed version of the training set. The perturbation is achieved by two randomization techniques—on one hand, each tree trains on a bootstrapped data set generated by drawing samples with replacement. On the other hand, at each node in a decision tree, only a subset of all features are considered as candidates for the next split. Tree randomization tends to reduce correlation among predictions of individual trees at the expense of increased variance of error estimates [61]. However, due to the averaging effect of ensembles of decorrelated models, it generally reduces the overall error variance of the ensemble RF [61]. That effect is partially negated by an increase in prediction bias, but broadly speaking the more decorrelated the trees the better the RF performance [61].

As each tree is constructed from a subsample of the training data, there is a tree-specific set of excluded, or out-of-bag (OOB) samples. The OOB predictions for the training set of a regression task are computed by walking each sample along the subset of trees in which that sample is OOB and averaging their predictions:

$$\begin{array}{cc} & \hat{f}\left(X_{i}\right)=\frac{1}{\left|OOB_{i}\right|}\sum_{t\in OOB_{i}}f^{t}\left(X_{i}\right)\\ & OOB_{i}=\left\{t:\in OOB\left(Tree_{t}\right);t=1\ldots T\right\}\text{,} \end{array}$$ (1)

where *X*_*i*_ are the input features associated with sample *i*, *OOB*_*i*_ is the set of trees in which sample *i* is OOB, *T* is the total number of trees in an RF and *f*^*t*^(*X*_*i*_) is the classification of the *i*^*th*^ sample in tree *t*. Similarly, for classification tasks the averaging of tree OOB predictions is replaced by majority vote.

It has been shown that the OOB error rate provides a very good approximation to generalization error [56]. For that reason, AKLIMATE uses RF kernels based on OOB predictions as level-one data for the MKL meta-learner training (see *RF Kernel* and *Stacked Learning* below).

### Kernel learning

Kernel learning is an approach that allows linear discriminant methods to be applied to problems with non-linear decision boundaries [62]. It relies on the “kernel trick”—a transformation of the input space to a different (potentially infinite dimensional) feature space where the train set samples are linearly separable. The utility of the “kernel trick” stems from the fact that the feature map between the input space and the feature space does not need to be explicitly stated—if the kernel function is positive definite (i.e. it has an associated Reproducing Kernel Hilbert Space), we only need to know the functional form of the feature space dot product in terms of the input space variables [62, 63]. A kernel function represents such a generalized dot product (**Note**: We define positive definiteness as in [62]—a kernel is positive definite if $\sum_{i=1}^{n}\sum_{j=1}^{n}c_{i}c_{j}K\left(x_{i},x_{j}\right)\;\geq \;0$ for $\forall x_{i},x_{j}\in X$ and $c_{1},\ldots ,c_{n}\in R$).

Commonly used kernel functions generally have an explicit closed form, such as a polynomial or a radial basis function. Kernel functions that encode more complex object relationships also exist, particularly if the objects can be defined in a recursive manner (ANOVA kernels, string kernels, graph kernels, see [64]). However, knowing the closed form kernel function is not a necessary condition—if the kernel positive definiteness can be verified and the kernel matrix of pairwise dot products (similarities) can be computed, we can still utilize the “kernel trick”. In fact, the Representer Theorem [62, 65] guarantees that the optimal solution to a large collection of optimization problems can be computed by kernel matrix evaluations on the finite-dimensional training data, ensuring the applicability of kernel-based alogirthms to real-world problems. More formally, for an arbitrary loss function *L*(*f*(*x*_1_)…*f*(*x*_*N*_)), the minimizer f* of the regularized risk function

$$\begin{array}{c} R\left(f\right)=L\left(f\left(x_{1}\right)\ldots f\left(x_{N}\right)\right)+{\Omega (\|f\|}_{H}^{2}) \end{array}$$ (2)

can be expressed as

$$\begin{array}{c} f^{*}=\sum_{i=1}^{N}\alpha_{i}k\left(x_{i},\right),\alpha_{i}\geq 0 \end{array}$$ (3)

provided that *k*(·, *x*) is a positive definite kernel and the regularization term $\Omega (\|f{\|}_{H}^{2})$ is a monotonically increasing function.

AKLIMATE uses the dependency structure of a RF trained on a (possibly multi-modal) feature set to define an implicit empirical kernel function. It then computes an RF kernel matrix (see *RF Kernel* below) of pairwise training set similarities to use in a kernel learning algorithm. As different feature sets can contribute complementary information for a given prediction task, AKLIMATE utilizes a Multiple Kernel Learning approach to integrate the available RF kernels into an optimal predictor.

### Multiple kernel learning

Kernel learning can lead to large improvements in accuracy, provided that an optimal kernel is selected. That choice can be difficult, however—the best kernel function for a multi-modal data set may be non-obvious, may entail tuning several hyperparameters, or may even lack a closed form expression. MKL addresses the problem of kernel selection by constructing a composite kernel that is a data-driven optimal combination of candidate kernels [50, 66–68]. Linear combinations of kernels of the form $K\left(\alpha \right)=\sum_{i=1}^{M}\alpha_{i}K_{i}$ with either a conical (∀*i*, *α*_*i*_ ≥ 0) or convex (∀*i*, *α*_*i*_ ≥ 0 and $\sum_{i=1}^{M}\alpha_{i}=1$) sum constraint on the kernel weights are by far the most commonly used because of their many desirable properties (although algorithms that allow non-linear combinations do exist—see [67]). Some important advantages are:

1. Conical/convex combinations of positive definite kernels are positive definite.
2. The composite kernel is associated with a feature space that is the concatenation of all individual kernel feature spaces.
3. The Representer Theorem is readily extendable to the the conical/convex combination case—the optimal solution f* takes the form $f^{*}=\sum_{m=1}^{M}\sum_{i=1}^{N}k_{m}\left(x,x_{i}\right)\alpha_{m,i}$ [51, 62].

MKL algorithms admit different forms of regularization depending on what norm of the kernel weights is chosen. AKLIMATE uses an elastic-net [45] regularizer on the norm of the individual kernel weights of the form $\lambda_{1}\sum_{i=1}^{M}\|\alpha_{m}{\|}_{K_{m}}+\lambda_{2}\sum_{i=1}^{M}{\left\|\alpha_{m}\right\|}_{K_{m}}^{2}$, where λ_1_, λ_2_ ≥ 0, *α*_*m*_ = (*α*_*m*,1_…*α*_*m*,*N*_)^*T*^, and $\|\alpha_{m}{\|}_{K_{m}}=\sqrt{\alpha_{m}K_{m}\alpha_{m}}$ is the definition of a kernel norm as in [51] and [69]. The elastic net regularization provides the flexibility to find both sparse and dense solutions with appropriately tuned λ’s—i.e. λ_1_ > > λ_2_ gives few non-zero kernel weights (sparse) while λ_1_ < λ_2_ gives many non-zero kernel weights (dense). Also note that λ_1_ = 0 and λ_2_ > 0 allows for a uniform kernel weight solution.

The explicit form of AKLIMATE’s optimization problem is:

$$\begin{array}{c} \underset{\alpha \in R^{NM},b\in R}{\text{min}}\sum_{i=1}^{N}L\left(y_{i},\sum_{m=1}^{M}\sum_{j=1}^{N}k_{m}\left(x_{i},x_{j}\right)\alpha_{m,j}+b\right)+\lambda_{1}\sum_{i=1}^{M}\|\alpha_{m}{\|}_{K_{m}}+\lambda_{2}\sum_{i=1}^{M}{\left\|\alpha_{m}\right\|}_{K_{m}}^{2} \end{array}$$ (4)

and is solved using the SpicyMKL algorithm described in [51]. The optimal kernel weights are recovered by:

$$\begin{array}{c} w_{m}=\{\begin{array}{cc} 0 & (\|\alpha_{m}^{*}{\|}_{K_{m}}=0),\\ \frac{\|\alpha_{m}^{*}{\|}_{K_{m}}}{\lambda_{1}+\lambda_{2}{\left\|\alpha_{m}^{*}\right\|}_{K_{m}}} & \left(\text{otherwise}\right) \end{array} \end{array}$$ (5)

where α* is the solution to Eq (4). The model weights are re-scaled to satisfy $\sum_{i=1}^{M}w_{i}=1$.

### RF kernel

The most common approach to kernel matrix evaluation is to specify an explicit data dependency model for the kernel function—e.g. polynomial, Gaussian, ANOVA or graph kernels [64]. However, choosing the dependency structure a priori can lead to lack of robustness, particularly when it is not obvious what the right dependency structure is. AKLIMATE uses a RF to create an empirical approximation of the true dependency model and to evaluate the (implicit) kernel function associated with it (the RF kernel). RF kernels are robust to overfitting, generalize well to new data, and facilitate the integration of signals across data types.

Defining a kernel through a RF is not a new idea—in fact the concept was introduced at the same time as RFs [70]. In particular, for a random forest of trees with an equal number of leaves and uniform predictions in each leaf, a positive definite kernel can be defined based on the probability two samples share a leaf [70]:

$$\begin{array}{c} K\left(x_{i},x_{j}\right)=\underset{M\to \infty }{\text{lim}}\frac{1}{M}\sum_{m=1}^{M}\sum_{t=1}^{T}I\left(i,j\in R_{t}\left(\theta_{m}\right)\right)\text{,} \end{array}$$ (6)

where *M* is the number of trees in the forest, *T* is the number of leaves in a tree, *θ*_*m*_ is a variable capturing the random selection of training set samples and features in the process of constructing the *m*th tree, *R*_*t*_ is the *t*th leaf of the *m*th tree, and *I*(⋅) is the indicator function. The finite approximation of Eq (6) (*M* < ∞; Fig 5, *K*_3_ kernel) is positive definite [71]. Kernels generated from RFs and their theoretical properties are also discussed in [72] and [73].

AKLIMATE’s RF kernel extends the original definition (Eq (6)) by incorporating two additional RF-derived statistics. The intuition behind the first one is that two samples predicted to have the same label within a tree should be considered alike even if they fall into different leaves (Fig 5, *K*_1_ kernel). The reasoning for the second one is that earlier node splits in a tree generally separate more distinct sample groups, while later splits tend to fine tune the decision boundary, highlighting more subtle differences. Thus, irrespective of the predicted labels, two samples that end up in different tree depths—one early- and one late-split leaf—should be considered less similar than samples landing in two late-split leaves (Fig 5, *K*_2_ kernel). Incorporating these three patterns, AKLIMATE’s RF kernel matrix is computed using the following steps:

1. Calculate similarity over predictions across RF trees:
  $$\begin{array}{c} K_{1}\left(x_{i},x_{j}\right)=\text{exp}\left(-\frac{\|p_{i}-p_{j}{\|}^{2}}{\sigma }\right)\text{,} \end{array}$$ (7)
  where *p*_*i*_ = (*p*_*i*,1_, *p*_*i*,2_, …, *p*_*i*, *M*_) is a vector of predictions for data point *i* from the *M* trees in the RF. *p*_*i*_ always has continuous entries—either the actual predictions in a regression setting, or the probabilities of class membership for classification problems. The ‖*p*_*i*_ − *p*_*j*_‖^2^ distances are divided by the scaling constant *σ* = max_*i*,*j*_‖*p*_*i*_ − *p*_*j*_‖^2^ so that they map to the [0, 1] range. Exponentiation of the negative distances converts them to similarities.
2. Compute similarity over leaf node indices across RF trees:
  $$\begin{array}{c} K_{2}\left(x_{i},x_{j}\right)=\text{exp}\left(-\frac{\|t_{i}-t_{j}{\|}^{2}}{\sigma }\right)\text{,} \end{array}$$ (8)
  where *t*_*i*_ = (*t*_*i*,1_, *t*_*i*,2_, …, *t*_*i*,*M*_) is a vector of the leaf indices of sample *i* across the RF. Tree nodes are indexed starting from the base node and then sequentially across each depth level of the tree. The scaling constant *σ* is set in the same manner as in *K*_1_.
3. Calculate similarity over leaf node co-occurrence using the finite approximation of Eq (6) with variable number of leaves per tree:
  $$\begin{array}{c} K_{3}\left(x_{i},x_{j}\right)=\text{exp}\left(\left(\frac{1}{M}\sum_{m=1}^{M}\sum_{t=1}^{T_{m}}I\left(i,j\in R_{t}\left(\theta_{m}\right)\right)\right)-1\right)\text{,} \end{array}$$ (9)
  where the exponential transformation is added to keep *K*_3_ on a similar scale to *K*_1_ and *K*_2_.
4. Calculate the RF kernel as the geometric mean of the element-wise product of *K*_1_,*K*_2_ and *K*_3_:
  $$\begin{array}{c} K\left(x_{i},x_{j}\right)=\sqrt[{3}]{K_{1}\left(x_{i},x_{j}\right)\circ K_{2}\left(x_{i},x_{j}\right)\circ K_{3}\left(x_{i},x_{j}\right)}\text{.} \end{array}$$ (10)

Importantly, since the components *K*_1_, *K*_2_, and *K*_3_ are positive definite, so too is *K*. *K*_1_ and *K*_2_ are Gaussian kernels over P×P,$P\subseteq R^{m}$ and T×T,$T\subseteq I^{m}$ respectively, which ensures their positive definiteness [64]. *K*_3_ is the exponent of a positive definite kernel, i.e. a positive definite kernel as well [64]. Finally, *K* is the Hadamard product of three positive definite kernels raised to a positive power—both of these operations preserve positive definiteness [64].

### Stacked learning

Stacked learning is a generalization of ensemble learning in which the stacked model (meta-learner) uses the prediction output of its components (base learners) as training data for the computation of the final predictions [57, 74]. What makes stacked learning unique is that the base learner predictions (level-one data) are generated in a cross-validated manner that excludes each sample from the training set that produces its predicted label. More specifically, if our training set (level-zero data) is $X=\{X_{i}:i=1\ldots N,X_{i}\in R^{p}\}$ with labels $Y=\{Y_{i}:i=1,\ldots ,N,Y_{i}\in R\}$ and we have a collection of base learners **Δ** = {Δ_1_, …, Δ_*S*_} then the stacked generalization proceeds as follows [59, 75]:

1. Randomly split the level-zero data into *V* folds of roughly equal size—*h*_1_, …, *h*_*V*_ (*V*-fold cross validation). Note that each *h*_*v*_ defines a set of indices that select a subset of the samples in *X*.
2. For each Δ_*s*_ base learner, train *V* models, collectively denoted as ${\hat{\Delta }}_{s}=\left\{{\hat{\Delta }}_{s,1}\ldots {\hat{\Delta }}_{s,V}\right\}$. Each model ${\hat{\Delta }}_{s,v}$ uses $X\backslash X_{h_{v}}$ to train on and generates predictions for $X_{h_{v}}$.
3. Concatenate the *V* sets of predictions from ${\hat{\Delta }}_{s}$ into a vector *z* of length *N*. The *N* × *S* matrix *Z* of such vectors for all *S* base learners becomes the feature matrix for level-one training.
4. Train a meta-learner Ψ on *Z* with hyperparameter tuning if necessary, again using *Y* as the labels.
5. The base learners used in the final stacked model are created using all training samples, so one final (non-cross-validated) training round is needed. To this end, train each Δ_*s*_ base learner on the full level-zero training set *X*.
6. Form the stacked model using the base-learners trained on the full data set and the meta-learner to obtain ({Δ_*s*_: *s* = 1, …, *S*}, Ψ). To predict on a new data point *X*_*new*_, compute a 1 × *S* vector *Z*_*new*_ = {Δ_*s*_(*X*_*new*_): *s* = 1, …, *S*} and use Ψ(*Z*_*new*_) as the stacked model’s prediction.

The predictive performance of the meta-learner is generally improved when the base learner predictions are maximally uncorrelated. This is achieved either by using different algorithms, or by varying the parameters of a particular modeling approach [58, 74]. AKLIMATE generates diversity through the use of feature subsets built around distinct biological concepts and processes.

#### Super learner

Stacked learning imposes no conditions on the choice of meta-learner Ψ. The disadvantage of such flexibility is the lack of theoretical results for the improved empirical performance of stacking. A Super Learner [58] is a type of stacked learner with restrictions on Ψ that give provable desirable properties. The main such constraint is that the optimal meta-learner Ψ* is the minimizer of a bounded loss function. A Super Learner for which {Δ_1_, …, Δ_*S*_} and Ψ have uniformly bounded loss functions exhibits the “oracle” property—Ψ* is asymptotically guaranteed to perform as well as the optimal base learner $\Delta_{s}^{*}$ under the true data-generating distribution [76, 77]. Furthermore, if we constrain the choice of Ψ to $\Psi =\sum_{i=1}^{S}\alpha_{i}\Delta_{i}$,∀*α*_*i*_ ≥ 0, Ψ* asymptotically converges to the performance of the optimal conical combination of {Δ_1_, …, Δ_*S*_} [58, 59]. This is the main reason why Ψ often takes the form of regularized linear or logistic regression.

For example, if the aim is to predict a continuous variable, one can set $\Psi =\sum_{i=1}^{S}\alpha_{i}{\hat{\Delta }}_{i}$ and solve the regression problem:

$$\begin{array}{c} \underset{\alpha }{\text{min}}\sum_{i=1}^{N}{\left(Y_{i}-\sum_{j=1}^{S}\alpha_{j}{\hat{\Delta }}_{j}\left(X_{i}\right)\right)}^{2}\text{,} \end{array}$$ (11)

which can be regularized or subjected to a convex sum constraint on the *α* weights (e.g. ∀*α*_*i*_ ≥ 0, $\sum_{i=1}^{S}\alpha_{i}=1$) [58, 59]. Similarly, the squared error loss of Eq (11) can be replaced with the logistic loss to solve a classification problem:

$$\begin{array}{c} \underset{\alpha }{\text{min}}\sum_{i=1}^{N}\log\left(1+\text{exp}\left(-Y_{i}*\left(\sum_{j=1}^{S}\alpha_{j}{\hat{\Delta }}_{j}\left(X_{i}\right)\right)\right)\right) \end{array}$$ (12)

maintaining all theoretical Super Learner results.

### AKLIMATE

To our knowledge, AKLIMATE is the first instance of a kernel-based stacked learner. The base learners {Δ_1_, …, Δ_*S*_} are RFs, each of which is used to produce an associated kernel. The meta-learner Ψ is an elastic-net regularized MKL that can be interpreted as the kernel learning counterpart of linear regression. Before describing the algorithm, we first discuss how the level-one RF kernels are constructed using OOB samples.

#### AKLIMATE level-one (OOB) kernel construction

AKLIMATE uses RF kernels as the level-one training data. Normally, level-one data is generated with cross-validation to help the meta-learner avoid overfitting. Analogously, AKLIMATE utilizes out-of-bag (OOB) samples to generate the components of the RF kernels. For each pair of samples in the RF, the kernel similarity matrix is calculated using only those trees for which both of the samples have been withheld, with the following procedure:

1. For a RF with *M* trees, define *OOB*(*m*) as the set of samples that are OOB in the *m*th tree. Let *I*_*OOB*_ be the tree-level indexing function recording when both samples *i* and *j* are simultaneously OOB in a given RF; i.e.:
  $$\begin{array}{cc} I_{OOB}\left(i,j\right) & =(\gamma_{1ij},\ldots ,\gamma_{mij})\text{,}\;\text{where}\\ \gamma_{mij} & =\{\begin{array}{cc} 1 & i,j\in OOB(m),\\ 0 & \text{otherwise} \end{array}\;\text{.} \end{array}$$ (13)
2. Compute the first constituent kernel matrix:
  $$\begin{array}{c} K_{1}\left(x_{i},x_{j}\right)=\text{exp}\left(-\frac{\|\left\langle p_{i},I_{OOB}\left(i,j\right)\right\rangle -\left\langle p_{j},I_{OOB}\left(i,j\right)\right\rangle {\|}^{2}}{\sigma }\right)\;\text{.} \end{array}$$ (14)
3. Compute the second constituent kernel matrix:
  $$\begin{array}{c} K_{2}\left(x_{i},x_{j}\right)=\text{exp}\left(-\frac{\|\left\langle t_{i},I_{OOB}\left(i,j\right)\right\rangle -\left\langle t_{j},I_{OOB}\left(i,j\right)\right\rangle {\|}^{2}}{\sigma }\right)\;\text{.} \end{array}$$ (15)
4. Compute the third constituent kernel matrix:
  $$\begin{array}{c} K_{3}\left(x_{i},x_{j}\right)=\\ \;\text{exp}(\left(\frac{1}{\sum I_{OOB}\left(i,j\right)}\sum_{m=1}^{M}\sum_{t=1}^{T_{m}}I\left(i,j\in R_{t}\left(\theta_{m}\right)\right)I_{OOB}{\left(i,j\right)}_{m}\right)-1)\;\text{.} \end{array}$$ (16)
5. Calculate the combined kernel matrix:
  $$\begin{array}{c} K\left(x_{i},x_{j}\right)=\sqrt[{3}]{K_{1}\left(x_{i},x_{j}\right)\circ K_{2}\left(x_{i},x_{j}\right)\circ K_{3}\left(x_{i},x_{j}\right)}\;\text{,} \end{array}$$ (17)

with the same notation and *σ* calculations as in Eqs (7)–(10).

AKLIMATE’s MKL meta-learner has two elastic-net hyperparameters (λ_1_, λ_2_) that require tuning. This is done by generating a random set of (λ_1_, λ_2_) pairs and ranking them based on *V*-fold (default *V* = 5) cross-validation fit with OOB RF kernels as input. To improve generalization, we use a simplified version of the overfit correction procedure in [78]—instead of selecting the hyperparameters that produce the best CV fit, we choose the ones corresponding to the 90th percentile of the distribution of the CV fit metric.

#### AKLIMATE algorithm

We next describe AKLIMATE’s learning algorithm. The method takes training data $\left(X,Y\right)=\{\left(X_{i},y_{i}\right):X_{i}=X_{i1}\cup \cdots \cup X_{id},i=1\ldots N,d=1\ldots D;y_{i}\subset Y\}$, with *X* a tensor of *N* samples and *D* data types with feature memberships $C={\left\{C_{i}\right\}}_{i=1}^{D}$ respectively, and training labels *Y*. In addition, *S* feature sets (e.g. pathways) are supplied, each containing a list of features *P*_*s*_ such that the complete set is $P={\left\{P_{s}\right\}}_{s=1}^{S}$. The algorithm outputs a meta-learner, Ψ* and a set of selected base learners Δ*. In addition, a user-supplied parameter *G* determines the number of top feature sets (pathways) to incorporate for each sample during the base learning step. We use *G* = 5 in practice.

Formally, AKLIMATE operates according to the pseudocode in Algorithm 1. First AKLIMATE trains a separate RF for each feature set using data from all modalities relevant to the features in the set. Model accuracy *τ* is stored so that the top *L* models (by *τ*) can be selected that correctly predict an individual sample. A final set of relevant RFs Δ* is obtained by taking the union over all sample-specific top *L* models. The helper function RFKERNELOOB creates a kernel from a given RF utilizing the out-of-bag approach described previously. MKL uses these level-one kernels to tune the elastic net hyperparameters λ_1_ and λ_2_ based on cross-validation performance. A final meta-learner Ψ* is then trained by running MKL with kernels constructed from the full RFs (RFKERNEL helper function) and with the determined elastic net hyperparameters.

**Algorithm 1**: AKLIMATE

Input: (*X*, *Y*), *C*, *P*, *G*

Output: Ψ*, Δ*

**for** *s* ∈ *S* **do**

 $\hat{P_{s}}\leftarrow \cup_{d=1}^{D}\left(P_{s}\cap C_{d}\right)$;        // list of available features

 $RF_{s}\leftarrow \text{RFTRAIN}\left(X_{\hat{P_{s}}},Y\right)$;      // Train respective base learner

 ${\hat{Y}}^{s}\leftarrow RF_{s}\left(X_{\hat{P_{s}}}\right)$;            // Compute OOB predictions

 $\tau_{s}\leftarrow \text{FIT}\left(Y,{\hat{Y}}^{s}\right)$;    // fit statistic based on OOB predictions

**end**

**for** *n* ∈ *N* **do**

 $\Delta_{n}^{*}\leftarrow {\left\{RF_{i_{g}}:RF_{i_{g}}\left(X_{n}\right)=Y_{n}\;\ldots \tau_{i_{1}}\geq \tau_{i_{2}}\cdots \geq \tau_{i_{g}}\geq \text{max}\left({\left\{\tau_{s}\right\}}_{s=1}^{S}\backslash \left\{\tau_{i_{1}},\ldots ,\tau_{i_{g}}\right\}\right)\right\}}_{g=1}^{G}$;   // pick top *G* (ranked by *τ*_*s*_) RFs predicting sample *n* correctly

**end**

$\Delta^{*}\leftarrow \cup_{n=1}^{N}\Delta_{n}^{*}$

*K*_*oob*_ ← {}

**for** *RF*_*r*_ ∈ Δ* **do**

 *K*_*oob*_ ← *K*_*oob*_ ∪ RFKERNELOOB(*RF*_*r*_, *X*)

**end**

$\left(\lambda_{1}^{*},\lambda_{2}^{*}\right)\leftarrow {\text{argmax}}_{\lambda_{1},\lambda_{2}}\left\{\text{CV(MKL}\left(K_{oob},\lambda_{1},\lambda_{2},Y\right)\right)\}$

*K*_*full*_ ← {}

**for** *RF*_*r*_ ∈ Δ* **do**

 *K*_*full*_ ← *K*_*full*_ ∪ RFKERNEL(*RF*_*r*_, *X*)

**end**

$\Psi^{*}\leftarrow \text{MKL}\left(K_{full},\lambda_{1}^{*},\lambda_{2}^{*},Y\right)$;      // Train MKL meta-learner

#### AKLIMATE selection of relevant RFs

AKLIMATE’s selection step for the best RFs Δ* in Algorithm 1 can filter the full collection of feature sets down to a subgroup of relevant sets two or more orders of magnitude smaller in size. This makes it possible to incorporate appreciably more feature sets than standard MKL algorithms which require kernel evaluation for all feature sets. When the number of such sets is in the thousands, MKL can be computationally very slow, even for data sets of small sample size. However, usually only a small proportion of the feature set collection is truly explanatory for a given prediction task. Thus, filtering out the non-relevant parts of the compendium does not impact MKL accuracy yet drastically improves computation time.

Algorithm 1 demonstrates the Δ* discovery process for a classification task. However, if *Y* is continuous (i.e. a regression problem), the predictions and labels are not directly comparable for equality. One approach is to take the squared error of the prediction-label differences and use that metric to re-rank RFs for each sample. In our experience, this leads to the selection of suboptimal RFs due to overfitting. Instead, AKLIMATE uses a more robust scheme that shows better results in practice—the vector of predictions for each sample across all RFs ${\left\{{\hat{Y}}_{n}^{s}\right\}}_{s=1}^{S}$ is binarized into matching and non-matching predictions and then the standard classification case selection rule is applied. The binarization is done as follows:

$$\begin{array}{c} {\hat{Y}}_{n_{bin}}^{s}=\{\begin{array}{cc} 1 & \;\text{if}\;\left|{\hat{Y}}_{n}^{s}-Y_{n}\right|\leq quantile\left({\left\{\left|{\hat{Y}}_{n}^{s}-Y_{n}\right|\right\}}_{s=1}^{S},q\right),\\ 0 & \;\text{otherwise} \end{array}\;\text{,} \end{array}$$ (18)

where *q* is a user-specified quantile of the empirical distribution of absolute prediction errors ${\left\{\left|{\hat{Y}}_{n}^{s}-Y_{n}\right|\right\}}_{s=1}^{S}$ (default *q* = 0.05). This setup prioritizes RFs that perform near-optimally on individual data points and optimally when the training set is considered as a whole.

#### AKLIMATE importance weighting of individual features and feature sets

Feature set weights *w*^*FS*^, $\sum_{i}w_{i}^{FS}=1$, are recovered directly from the optimal MKL meta-learner Ψ*(Eq (5)). Feature weights can be calculated as $w_{i}^{F}=\sum_{k\in {\hat{P(\cdot )}}_{i}}w_{k}^{FS}m\left(RF_{k},i\right)$ where ${\hat{P(\cdot )}}_{i}=\left\{\hat{P_{s}}:i\in \hat{P_{s}}\;\&\;RF_{\hat{P_{s}}}\in \Delta^{*},s=1,\ldots ,S\right\}$ is the set of all feature sets that have feature *i* as its member and their associated RF was selected among the set of best RFs Δ*, while *m*(*RF*_*k*_, *i*) is an *RF*-specific feature importance score computed from the RF associated with the *k*th such feature set.

The simplest way to compute *m*(*RF*_*k*_, *i*) is by averaging the improvement in the splitting criterion over all nodes that used feature *i* as a splitting variable. For the often used Gini impurity measure, this involves computing the mean difference in impurity before and after each split, with larger mean impurity decreases indicative of more important variables [79]. While fast, this rule suffers from important shortcomings—for example, it is biased in favor of variables with more potential split points (e.g. continuous or categorical with a large number of categories), particularly when trees train on bootstrapped data [80].

Many alternative *m*(*RF*_*k*_, *i*) rules have been proposed [80–82]. For our work, we choose as default the permutation-based importance calculation described in the original RF paper [56]—the vector of measurements for feature *i* is randomly permuted and the permuted variable is used in the calculation of OOB predictions; the difference in error rate between the permuted and non-permuted OOB predictions is taken as a measure of feature *i*’s importance. Permutation-based importance is robust and generally performs on par with more complex *m*(*RF*_*k*_, *i*) rules. Its biggest drawback is the higher computational cost. In cases where compute time is the main constraint, we recommend the actual impurity reduction (AIR) metric [83], which is an extension of the pseudodata-augmented approach in [81]. It is similar in speed to the Gini impurity importance, but retains the desirable properties of permutation-based methods. While AIR can lead to a small prediction accuracy penalty, in our experience this effect has been negligible for classification tasks (for regression problems we still recommend permutation-based importance).

## Supporting information

S1 Text Technical details of the experiments and the implementation of the AKLIMATE algorithm. (PDF)

S1 Table Most informative feature sets for breast cancer survival prediction in METABRIC data. AKLIMATE weights were averaged over 50 train/test splits. The table lists the 20 most relevant feature sets, out of 1836 feature sets with a non-zero weight in at least one train/test split. Weights were normalized to sum to 1. Aliases for the top 10 feature sets are included in brackets—they provide a more descriptive name for the underlying biological function, based on information gathered from the source publication. The aliases are used in Fig 2 and throughout the text.
(PDF)

S1 Fig AKLIMATE versus alternative ways of ensembling/stacking component RFs. To ensure fair comparison, the component RF set for each prediction task was restricted to AKLIMATE’s best RFs Δ*. Ensemble superlearner component RFs—learning a regularized linear regression on predictions from component RFs; ensemble average component RFs—taking the average of the predictions of component RFs; top component RF—only using predictions from the top ranked RF. (A) UCEC TCGA MSI prediction. (B) METABRIC Breast Cancer survival prediction. (C) Achilles shRNA knockdown prediction. P-values for paired Wilcoxon signed rank test.
(EPS)

S2 Fig AKLIMATE results for the AKLIMATE-reduced model (using only 196 PID pathways) on UCEC TCGA cohort. Top 10 predictive feature sets and top 50 predictive features from 50 stratified 75% train/25% test splits are shown. Organized as Fig 1.
(EPS)

S3 Fig AKLIMATE performance on predicting MSI-High vs MSI-Low+MSS in TCGA COADREAD cohort. AUC computed for 50 75%/25% stratified train/test splits. P-values for paired Wilcoxon signed rank test. Methods as in Fig 1. Mean AUROCs of 0.99±0.011, 0.983±0.014, 0.976±0.022, 0.988±0.015, 0.981±0.027, 0.989±0.015 for aklimate, aklimate-reduced, sbmkl, dbmkl, sbmtmkl, and dbmtmkl respectively.
(EPS)

S4 Fig Predictive accuracy for selected methods on METABRIC dataset. Methods include 3 versions of AKLIMATE with different combinations of input data types and some high-performing competitive methods (see main text). Point-and-whiskers plot displays mean and standard error of a method’s test accuracy during cross-validation. Mean and standard error for FSMKL and BCC taken from [16].
(EPS)

S5 Fig AKLIMATE accuracy on METABRIC survival prediction for models restricted to KEGG pathways as feature sets. (EPS)

S6 Fig BRCA subtype-specific prediction accuracy on METABRIC survival prediction for AKLIMATE models using expression, copy number, and clinical features. Samples were assigned to BRCA subtypes using the PAM50 gene expression signature [25].
(EPS)

S7 Fig Method performance on predicting cell line viability after shRNA gene knockdowns. A) Measured by Spearman correlation. B) Measured by the number of times an algorithm produced the best Spearman correlation on a prediction task. C) Measured by Pearson correlation. D) Measured by the number of times an algorithm produced the best Pearson correlation on a prediction task.
(EPS)

S8 Fig RMSE scores for 37 shRNA knockdown prediction tasks. Rows correspond to individual gene knockdowns; columns represent different methods. To highlight differential performance within each task, rows are centered by subtracting the median. Lower centered scores represent lower RMSE (better performance).
(EPS)

S9 Fig Data type importance proportions for 37 shRNA knockdown prediction tasks. Columns correspond to the relative contributions of each input data type across prediction tasks. Relative contributions were computed by summing the model-assigned feature importance scores within individual data types. Columns are normalized to sum to 1.
(EPS)

S10 Fig AKLIMATE results highlighting the 10 most informative feature sets and 50 most informative features for the task of predicting FOXA1 shRNA knockdown viability. Organized as Fig 1.
(EPS)

S11 Fig AKLIMATE results highlighting the 10 most informative feature sets and 50 most informative features for the task of predicting KRAS shRNA knockdown viability when no mutation features are used. Feature and feature set weights averaged over 10 matched stratified 80%/20% train/test splits. Organized as Fig 1.
(EPS)

S12 Fig Metrics for PIK3CA AKLIMATE models with and without the use of mutational profiles for 8 key regulators. Results averaged over 10 matched stratified 80%/20% train/test splits.
(EPS)

S13 Fig Metrics for CTNNB1 AKLIMATE models with and without the use of mutational profiles for 8 key regulators. Results averaged over 10 matched stratified 80%/20% train/test splits.
(EPS)
