# Supplementary material for: Accurate cancer phenotype prediction with AKLIMATE, a stacked kernel learner integrating multimodal genomic data and pathway knowledge
Source: PLoS Comput Biol. 2021 Apr 16;17(4):e1008878. doi: 10.1371/journal.pcbi.1008878 (PMC8081343; doi:10.1371/journal.pcbi.1008878)
Supplement: S1 Text — (PDF) [file pcbi.1008878.s001.pdf]

Supporting Text

Data Acquisition

Microsatellite Instability

Data for the COADREAD and UCEC TCGA cohorts were downloaded from the Synapse copy of the PANCAN12 TCGA cohort [1] (synapse object id syn300013, `https://www.synapse.org/#!Synapse:syn300013/wiki/70804`). The UCEC RNA-Seq upper-quantile normalized RSEM values (syn1446289, see [1]) were log transformed and used as the basis for BMKL- (see [2]) and AKLIMATE-specific filtering (see *AKLIMATE pre-processing*). The full expression data set contained 20,501 features. The filtered set used by AKLIMATE was reduced to 13,424 features. The MSI status for UCEC patients was extracted from UCEC clinical data (syn1446167).

Similarly, the COADREAD expression dataset was created by joining the COAD (syn1446197) and READ (syn1446276) PANCAN12 cohorts, log transforming the combined matrix and applying respective filtering steps. The AKLIMATE filtered data set contained 14,036 features. MSI status was downloaded from Firebrowse (`http://gdac.broadinstitute.org/runs/stddata__2016_01_28/data/COADREAD/20160128/gdac.broadinstitute.org_COADREAD.Clinical_Pick_Tier1.Level_4.2016012800.0.0.tar.gz`) and matched to the whitelisted samples for the joint COADREAD PANCAN12 expression set.

METABRIC Survival

Expression (Illumina HT12 array), copy number (Affymetrix SNP 6.0) and clinical data for the METABRIC cohort [3] were downloaded from `https://www.synapse.org` (Synapse ID syn1688369). Expression and copy number data were processed as described in the marker paper [3]. Expression data used Illumina HT12V3 probe identifiers while copy number data had Entrez gene features. Since our pathway compendium was HGNC-based, we translated each HGNC gene set to all Illumina probes and Entrez gene IDs matching any of its members. We used the *IlluminaHumanv4.db* Bioconductor package for the Illumina-HGNC map and the *org.Hs.eg.db* package for the Entrez-HGNC map.

To match the analysis in [4], we restricted the METABRIC cohort to 639 patients (list obtained in personal communication with authors). For the same reason, we did not use the full set of clinical information available, but limited it to variables used in [4], namely:

1. Age at diagnosis
2. Tumor size
3. Tumor grade
4. Tumor stage
5. Number of positive lymph nodes
6. Histological type
7. Estrogen receptor IHC status and expression-based status
8. HER2 IHC status, SNP6 status, and expression-based status
9. Nottingham Prognostic Index

10. PAM50-based breast cancer subtype
11. Cellularity
12. Composite treatment status

The last clinical variable was not present in the METABRIC clinical file, but was created by integrating aspects of other clinical variables in a manner described in [4].

Expression and copy number data sets were filtered as described in *AKLIMATE pre-processing*, with mean and variance calculations based on the reduced rather than the full cohort. The combined AKLIMATE filtered feature set contained 20,022 expression, 8608 copy number and 15 clinical features.

Accuracies for FSMKL and BCC methods were taken from [4].

shRNA Knockdown Profiles

Achilles 2.4.3 shRNA knockdown profiles were downloaded from `https://depmap.org`. The data release contained ATARiS [5] gene-level profiles for 5711 genes across 216 CCLE cell lines. Each profile was computed by aggregating the profiles of multiple shRNAs targeting an individual gene. ATARiS was run with a threshold of $p = 0.05$ on the samples and shRNAs that passed QC inspection (see online QC manifest of the Achilles 2.4.3 data). The mutation profiles of 8 regulators (KRAS, NRAS, PIK3CA, BRAF, PTEN, APC, CTNNB1 and EGFR) were extracted from the sample annotation file for the Achilles 2.4.3 data release.

Matching expression and copy number characterizations of individual cell lines were downloaded from the Cancer Cell Line Encyclopedia (CCLE, `https://portals.broadinstitute.org/ccle/data`). Expression was measured using Affymetrix U133 Plus 2.0 array, aggregated via Robust Multi-array Average and quantile normalized (see 2012 expression data release on CCLE website). Copy number was evaluated with Affymetrix SNP 6.0 arrays and segmentation of the normalized log2 probe ratios via Circular Binary Segmentation (see 2012 copy number release on CCLE website). GISTIC2 version 2.0.22 was run on the copy number data with default parameters (amplification and deletion thresholds of 0.1, broad event threshold of 0.7, enabled arm level peel-off events, and gene collapsing set to "extreme"). Hierarchical VIPER (hVIPER) was run on the expression data as described in [6] and [7].

Expression (18,900 features), copy number (23,316 features), GISTIC (24,924 features) and hVIPER activity (447 features) data types were provided to all methods for method-specific preprocessing (see main text). In case no pre-processing was specified, a method used all available features.

AKLIMATE used the filtering steps described in *AKLIMATE pre-processing.* The combined AKLIMATE filtered feature set contained 13,652 expression, 10,086 copy number, 9,557 GISTIC and 447 hVIPER activity features.

Feature Sets

We used four main sources for biologically relevant feature sets:

1. C2 (Curated Gene Sets) and C5 (Go Gene Sets) collections of MSigDB [8].
2. The GeneSigDB curated collection of published signatures [9].
3. Pathway Commons [10]- a database of databases covering the spectrum of metabolic, molecular, signaling, regulatory and genetic interactions.

4. Gene sets related to chromosomal location. These were constructed by passing TCGA LIHC segmented copy number data through the *CNRegions* function of the *iClusterPlus* [11] R package, with $\epsilon = 0.0025$.

We excluded gene sets from the Small Molecular Pathway Database [12] (SMPDB, part of Pathway Commons) due to the high redundancy and small size of many of the signatures. The "canonical" pathways in the MSigDB C2_CP subcollection were also removed due to their high degree of overlap with pathways contained in the more extensive Pathway Commons resource. Finally, all sets with more that 1000 members were removed to maintain specificity. The final compendium consisted of 17,273 gene sets with median size of 30 (min size of 1, max size of 991).

AKLIMATE pre-processing

Data for all three case studies was processed in the following manner:

1. Expression data was filtered based on the mean and variance of genes across samples - any gene whose mean or variance falls in the bottom 20% of the mean/variance empirical distribution was discarded.

2. Similar to expression, copy number data, if available, was filtered using a cutoff set at 50%. Whenever GISTIC2 [13] discretized gene-level copy number calls were used they were first filtered in the same manner.

3. The 447 protein activity scores from hVIPER [6, 7] representing transcription factor and kinase regulator features were not filtered.

To speed up computation in the larger cohorts, expression and copy number filtered data for the two classification tasks (MSI and METABRIC survival prediction) were discretized by computing the quintiles of the distribution of each molecular feature and binning each quintile into a separate category. Finally, unordered categorical features (e.g. METABRIC clinical variables) were one-hot encoded prior to use by AKLIMATE.

AKLIMATE hyperparameters

AKLIMATE was run with the same gene set collection across all prediction tasks (see *Feature Sets*). To increase robustness, gene sets that had fewer than 15 features across all considered data modalities were discarded. Since different case studies use a different number of data types, this thresholding causes the number of eligible gene sets to be task-specific.

The same AKLIMATE hyperparameters were used across all case studies, except for minor deviations described in the main text. To reduce computation time, AKLIMATE component RFs were trained with 50% sampling without replacement - i.e. each tree was grown on a randomly selected 50% subsample of the training set. Studies have shown that this setup performs as well as bootstrapping in predictive accuracy benchmarks [14]. In addition, the trees in each RF base model were set to have minimum leaf size equal to 1% of the size of the training cohort. For the selection of the best RF models $\boldsymbol{\Delta}^*$, each RF contained 500 trees. Prior to kernel construction, the forests of $\boldsymbol{\Delta}^*$ are re-grown with 2000 trees each. Higher number of trees and smaller leaf size tend to provide better approximation to the class discrimination boundary, as demonstrated in [15].

It is generally recommended to keep *mtry* low- e.g. $\sqrt{P}$ (P - total number of features) for classification and $\frac{P}{3}$ for regression problems because decorrelation among the predictions of individual trees often leads to an improved performance of the ensemble RF (see *Methods*). AKLIMATE, however, is an ensemble of ensembles -

decorrelation can also be achieved by selecting component RFs that describe orthogonal gene sets. As a consequence, we can prioritize bias reduction within individual RFs - we recommend *mtry* values in the 25-75% range. In our experience, a setting of 25% is fast and accurate. Thus, the number of features randomly selected to try at each node (*mtry*) was set to 25% of the size of the queried gene set.

Finally, we used two different importance metrics for feature relevance evaluation - Actual Impurity Reduction (AIR) [16] for classification tasks (microsatellite instability and METABRIC survival), and permutation analysis [17] for regression problems (shRNA knockdown viability). See *Methods* for more details.

Implementation

We used the R package *ranger* [18] for calculations involving AKLIMATE's base RF learners, including permutation-/AIR-based variable importance. We chose *ranger* because of its flexibility in handling splitting rules, variable importance approaches, and learning tasks. It is also one of the fastest RF implementations currently available in R, particularly in problems where the number of features is much larger than the number of data points.

For our MKL learner, we ported *SpicyMKL* [19] to R. We chose *SpicyMKL* because its guaranteed super-linear convergence makes it possible to handle thousands of kernels. Furthermore, *SpicyMKL*'s elastic-net regularization allows maximum flexibility in terms of the number of kernels included in the optimal solution. Our R implementation of *SpicyMKL* called *SPICER* is available at `https://github.com/VladoUzunangelov/SPICER`.

Finally, an R package for AKLIMATE is available at `https://github.com/VladoUzunangelov/aklimate` with toy examples from the Stuart lab Wiki site at `https://sysbiowiki.soe.ucsc.edu/aklimate`. Running ALKIMATE does require a substantial amount of computational time and memory. Adding extra optimization steps to find parameters and using additional feature sets (pathways) for prediction, increase the running time but the trade-off to obtain biological insights for interpretation may be worthwhile. Learning the Random Forest model usually drives the running time and so one can expect the running time to scale linearly with the number of pathways included for analysis. The space requirements scale as $O(N^2)$, where N is the number of samples, due to the kernel construction step. A parallelized version of the code is provided in the github repository that allows a user to take advantage of multi-core setups.

We recorded the per task running times and space requirements for each of the experiments conducted for this study on our Xeon(R) CPU E7-8870 v4 (2.10GHz) machines using the code we provide in the AKLIMATE Docker image. For the UCEC and METABRIC experiments, each involved a single overall task (i.e. predicting MSI for UCEC or predicting patient outcome for METABRIC), while for the ACHILLES dataset, there was a task for each of the shRNA knockdowns (37 tasks overall reported in our study). Of course, performing cross-validation also increases the number of tasks (one for every cross-validation fold). Per task, the parallelized version of AKLIMATE ran in 7 minutes for UCEC using 30 cores and 90 GB of max RAM, 35 minutes for METABRIC using 25 cores and 223 GB of max RAM, and 40 minutes on the ACHILLES data (MDM4 shRNA as an example task) using 30 cores and 18 GB of max RAM. As a comparison, using a single core on the same machine resulted in a running time of 12.5 hours and a space requirement of 10GB of max RAM for the UCEC dataset. Further speedups are possible (e.g. by as much as 30% in our experience) by using optimized linear algebra libraries such as IntelMKL and openBLAS. The Docker container we provide includes default R libraries to make the code more portable and lends itself to an easier initial setup.

References

1. Hoadley KA, Yau C, Wolf DM, Cherniack AD, Tamborero D, Ng S, et al. Multiplatform Analysis of 12 Cancer Types Reveals Molecular Classification within and across Tissues of Origin. Cell. 2014;158(4):929–944. doi:10.1016/j.cell.2014.06.049.

2. Gönen M. Integrating gene set analysis and nonlinear predictive modeling of disease phenotypes using a Bayesian multitask formulation. BMC Bioinformatics. 2016;17(16):0. doi:10.1186/s12859-016-1311-3.

3. Curtis C, Shah SP, Chin SF, Turashvili G, Rueda OM, Dunning MJ, et al. The genomic and transcriptomic architecture of 2,000 breast tumours reveals novel subgroups. Nature. 2012;486(7403):346–352. doi:10.1038/nature10983.

4. Seoane JA, Day INM, Gaunt TR, Campbell C. A pathway-based data integration framework for prediction of disease progression. Bioinformatics. 2014;30(6):838–845. doi:10.1093/bioinformatics/btt610.

5. Shao DD, Tsherniak A, Gopal S, Weir BA, Tamayo P, Stransky N, et al. ATARiS: Computational quantification of gene suppression phenotypes from multisample RNAi screens. Genome Research. 2013;23(4):665–678. doi:10.1101/gr.143586.112.

6. Uzunangelov VJ. Prediction of cancer phenotypes through the integration of multi-omic data and prior information. Ph.D. Thesis, UC Santa Cruz. 2019. Available from: `https://escholarship.org/uc/item/5cs2x2bz`.

7. Robertson AG, Shih J, Yau C, Gibb EA, Oba J, Mungall KL, et al. Integrative Analysis Identifies Four Molecular and Clinical Subsets in Uveal Melanoma. Cancer Cell. 2017;32(2):204–220.e15. doi:10.1016/j.ccell.2017.07.003.

8. Subramanian A, Tamayo P, Mootha VK, Mukherjee S, Ebert BL, Gillette MA, et al. Gene set enrichment analysis: A knowledge-based approach for interpreting genome-wide expression profiles. Proceedings of the National Academy of Sciences of the United States of America. 2005;102(43):15545–15550. doi:10.1073/pnas.0506580102.

9. Culhane AC, Schröder MS, Sultana R, Picard SC, Martinelli EN, Kelly C, et al. GeneSigDB: a manually curated database and resource for analysis of gene expression signatures. Nucleic Acids Research. 2012;40(Database issue):D1060–D1066. doi:10.1093/nar/gkr901.

10. Cerami EG, Gross BE, Demir E, Rodchenkov I, Babur O, Anwar N, et al. Pathway Commons, a web resource for biological pathway data. Nucleic Acids Research. 2011;39(Database issue):D685–D690. doi:10.1093/nar/gkq1039.

11. Mo Q, Wang S, Seshan VE, Olshen AB, Schultz N, Sander C, et al. Pattern discovery and cancer gene identification in integrated cancer genomic data. Proceedings of the National Academy of Sciences. 2013;110(11):4245–4250. doi:10.1073/pnas.1208949110.

12. Jewison T, Su Y, Disfany FM, Liang Y, Knox C, Maciejewski A, et al. SMPDB 2.0: big improvements to the Small Molecule Pathway Database. Nucleic Acids Research. 2014;42(Database issue):D478–484. doi:10.1093/nar/gkt1067.

13. Mermel CH, Schumacher SE, Hill B, Meyerson ML, Beroukhim R, Getz G. GISTIC2.0 facilitates sensitive and confident localization of the targets of focal somatic copy-number alteration in human cancers. Genome Biology. 2011;12(4):R41. doi:10.1186/gb-2011-12-4-r41.

14. Friedman JH. Stochastic gradient boosting. Computational Statistics & Data Analysis. 2002;38(4):367–378. doi:10.1016/S0167-9473(01)00065-2.

15. Cao H, Bernard S, Sabourin R, Heutte L. Random forest dissimilarity based multi-view learning for Radiomics application. Pattern Recognition. 2019;88:185–197. doi:10.1016/j.patcog.2018.11.011.

16. Nembrini S, König IR, Wright MN, Valencia A. The revival of the Gini importance? Bioinformatics. 2018;doi:10.1093/bioinformatics/bty373.

17. Breiman L. Random Forests. Machine Learning. 2001;45(1):5–32. doi:10.1023/A:1010933404324.

18. Wright MN, Ziegler A. ranger: A Fast Implementation of Random Forests for High Dimensional Data in C++ and R. Journal of Statistical Software. 2017;77(1):1–17. doi:10.18637/jss.v077.i01.

19. Suzuki T, Tomioka R. SpicyMKL: a fast algorithm for Multiple Kernel Learning with thousands of kernels. Machine Learning. 2011;85(1-2):77–108. doi:10.1007/s10994-011-5252-9.
