# Supplementary material for: Accurate cancer phenotype prediction with AKLIMATE, a stacked kernel learner integrating multimodal genomic data and pathway knowledge
Source: PLoS Comput Biol. 2021 Apr 16;17(4):e1008878. doi: 10.1371/journal.pcbi.1008878 (PMC8081343; doi:10.1371/journal.pcbi.1008878)
Supplement: S1 Table — AKLIMATE weights were averaged over 50 train/test splits. The table lists the 20 most relevant feature sets, out of 1836 feature sets with a non-zero weight in at least one train/test split. Weights were normalized to sum to 1. Aliases for the top 10 feature sets are included in brackets—they provide a more descriptive name for the underlying biological function, based on information gathered from the source publication. The aliases are used in Fig 2 and throughout the text. (PDF) [file pcbi.1008878.s002.pdf]

feature sets	weights
GENESIGDB_BREAST_CREIGHTON09_594GENES (BREAST CANCER ER-/PR- DN)	0.0097
SCHAEFFER_PROSTATE_DEVELOPMENT_48HR_UP (PROSTATE DEVELOPMENT 48 HRS UP)	0.0093
KOINUMA_TARGETS_OF_SMAD2_OR_SMAD3 (SMAD2/3 TARGETS)	0.0089
GENESIGDB_LEUKEMIA_MARCUCCI08_696GENES (MIRNA TARGETS IN CYTOGENETICALLY NORMAL AML)	0.0075
NUYTTEN_NIPP1_TARGETS_DN (NIPP1 TARGETS DN)	0.0067
GENESIGDB_BREAST_MILLER05_P53 (BREAST CANCER P53 REGULOME)	0.0066
GENESIGDB_LYMPHOMA_LAM08_1502GENES (LYMPHOMA IL-6 AND IL-10 SIGNALING THROUGH STAT3)	0.0064
GOBERT_OLIGODENDROCYTE_DIFFERENTIATION_UP (OLIGODENDROCYTE DIFFERENTIATION UP)	0.0056
NEUTROPHIL_DEGRANULATION_REACTOME (NEUTROPHIL DEGRANULATION)	0.0053
GENESIGDB_BREAST_CREIGHTON08_772GENES (BREAST CANCER HER2 ENDOCRINE THERAPY RESISTANCE)	0.0051
GENESIGDB_BREAST_BARRY10_1022GENES	0.0048
CASORELLI_ACUTE_PROMYELOCYTIC_LEUKEMIA_DN	0.0046
MARKEY_RB1_ACUTE_LOF_DN	0.0046
GO_CELL_CYCLE_PHASE	0.0045
GENESIGDB_BREAST_YOSHIHARA10_88GENES	0.0043
GO_MITOTIC_CELL_CYCLE	0.0042
SMID_BREAST_CANCER_BASAL_DN	0.0041
GENESIGDB_OVARIAN_BARANOVA06_907GENES_SERTOLILEYDIG	0.0041
DUTERTRE_ESTRADIOL_RESPONSE_24HR_UP	0.0039
SARRIO_EPITHELIAL_MESENCHYMAL_TRANSITION_UP	0.0038

S1 Table. Most informative feature sets for breast cancer survival prediction in METABRIC data. AKLIMATE weights were averaged over 50 train/test splits. The table lists the 20 most relevant feature sets, out of 1836 feature sets with a non-zero weight in at least one train/test split. Weights were normalized to sum to 1. Aliases for the top 10 feature sets are included in brackets - they provide a more descriptive name for the underlying biological function, based on information gathered from the source publication. The aliases are used in Fig 2 and throughout the text.
